# Immunogenicity of personalized dendritic-cell therapy in HIV-1 infected individuals under suppressive antiretroviral treatment: *interim* analysis from a phase II clinical trial

**DOI:** 10.1186/s12981-021-00426-z

**Published:** 2022-01-12

**Authors:** Marcella Vassão de Almeida Baptista, Laís Teodoro da Silva, Sadia Samer, Telma Miyuki Oshiro, Iart Luca Shytaj, Leila B. Giron, Nathalia Mantovani Pena, Nicolly Cruz, Gisele Cristina Gosuen, Paulo Roberto Abrão Ferreira, Edécio Cunha-Neto, Juliana Galinskas, Danilo Dias, Maria Cecilia Araripe Sucupira, Cesar de Almeida-Neto, Reinaldo Salomão, Alberto José da Silva Duarte, Luís Mário Janini, James R. Hunter, Andrea Savarino, Maria Aparecida Juliano, Ricardo Sobhie Diaz

**Affiliations:** 1 Retrovirology Laboratory, Federal University of Sao Paulo, R. Pedro de Toledo, 669, Sao Paulo, SP 04039-032 Brazil; 2 LIM 56, Hospital das Clinicas HCFMUSP, Faculdade de Medicina, Universidade de Sao Paulo, Rua, Av. Dr. Enéas Carvalho de Aguiar, 255 - Cerqueira César, Sao Paulo, SP 05403-000 Brazil; 3 Department of Cell and Developmental Biology, Northwestern University, 300 E Superior St, Chicago, IL 60611 USA; 4 The Wistar Institute, 3601 Spruce St, Philadelphia, PA 19104 USA; 5 Departamento de Aféreses, Fundação Pró-Sangue Hemocentro de São Paulo, São Paulo, Brazil; 6 Disciplina de Ciências Médicas, Faculdade de Medicina da Universidade de São Paulo, São Paulo, Brazil; 7 Department of Infectious Diseases, Italian Institute of Health, Viale Regina Elena, 299, 00161 Rome, Italy

**Keywords:** Dendritic-cell therapy, HIV GAG, HLA Haplotypes, HIV cure research, Precision medicine

## Abstract

**Background:**

We developed a personalized Monocyte-Derived Dendritic-cell Therapy (MDDCT) for HIV-infected individuals on suppressive antiretroviral treatment and evaluated HIV-specific T-cell responses.

**Methods:**

PBMCs were obtained from 10 HIV^+^ individuals enrolled in trial NCT02961829. Monocytes were differentiated into DCs using IFN-α and GM-CSF. After sequencing each patient’s HIV-1 Gag and determining HLA profiles, autologous Gag peptides were selected based on the predicted individual immunogenicity and used to pulse MDDCs. Three doses of the MDDCT were administered every 15 days. To assess immunogenicity, patients’ cells were stimulated in vitro with autologous peptides, and intracellular IL-2, TNF, and interferon-gamma (IFN-γ) production were measured in CD4^+^ and CD8^+^ T-cells.

**Results:**

The protocol of ex-vivo treatment with IFN-α and GM-CSF was able to induce maturation of MDDCs, as well as to preserve their viability for reinfusion. MDDCT administration was associated with increased expression of IL-2 in CD4^+^ and CD8^+^ T-cells at 15 and/or 30 days after the first MDDCT administration. Moreover, intracellular TNF and IFN-γ expression was significantly increased in CD4^+^ T-cells. The number of candidates that increased in vitro the cytokine levels in CD4^+^ and CD8^+^ T cells upon stimulation with Gag peptides from baseline to day 15 and from baseline to day 30 and day 120 after MDDCT was significant as compared to Gag unstimulated response. This was accompanied by an increasing trend in the frequency of polyfunctional T-cells over time, which was visible when considering both cells expressing two and three out of the three cytokines examined.

**Conclusions:**

MDDC had a mature profile, and this MDDCT promoted in-vitro T-cell immune responses in HIV-infected patients undergoing long-term suppressive antiretroviral treatment.

*Trial registration* NCT02961829: (Multi Interventional Study Exploring HIV-1 Residual Replication: a Step Towards HIV-1 Eradication and Sterilizing Cure, https://www.clinicaltrials.gov/ct2/show/NCT02961829, posted November 11th, 2016)

**Supplementary Information:**

The online version contains supplementary material available at 10.1186/s12981-021-00426-z.

## Introduction

The onset of HIV-1 infection induces a robust immune response by the host [1, 2]. However, this response does not typically lead to immunological control of the infection, as shown by the rapid rebound of viremia after interruption of antiretroviral treatment (ART) [3–7]. Of note, the extent of post-therapy viral load rebound is proportional to the size of the HIV-infected cellular reservoir, and the size of this reservoir is in turn proportional to the length of the period during which the infection progresses without ART [7].

Pivotal for the immune response, dendriticcells (DC) are professional antigen presenters. These cells are anatomically positioned as sentinels for detecting danger signals, thus triggering an innate and adaptive immune response [8]. DCs can migrate to lymph nodes and other HIV-1 sanctuaries and, once activated, produce interleukins such as IL-12, IL-15, and IL-18, promoting a potent cytotoxic T-cell response necessary for the elimination of infected cells. Due to these characteristics, therapy approaches based on DCs have been proposed and tested to modulate the host’s immune response to HIV-1 infection [3, 9–20].

DC-based therapy relies on in-vitro pulsing or electroporating of autologous monocyte-derived DCs (MDDCs) with whole inactivated HIV-1, with RNA coding for viral antigens [12], or with specific sets of peptides [9, 11], to induce recognition of viral epitopes by the individual’s human leukocyte antigens (HLAs) on the DC surface. Peptide-pulsed DCs are then reinfused into the donor to provide an appropriate antigenic stimulus within the body when retroviral replication is still suppressed by ART. While MDDC therapy (MDDCT) has been typically administered to HIV-infected individuals under ART, the virus used for MDDCT design or preparation was obtained from samples of treatment-naive individuals. This choice was determined by the presence of a high percentage of defective viruses in cells of patients under long-term ART, rendering difficult the isolation and *in-vitro* expansion of high amounts of replication-competent HIV-1 [21, 22]. HIV-1 peptides typically employed in MDDCT design have been derived from highly conserved proteins of HIV-1, i.e., Gag, Pol, and Nef [9, 11]. Moreover, conserved portions of variable antigens such as Env have been considered [14, 15]. After infusion, the therapeutic efficacy of the MDDCT is assessed by interrupting ART to check whether viral load suppression can be maintained in the absence of drugs [10, 11, 15, 18, 19]. Moderate post-therapy efficacy was observed in some of these studies [10, 18]. In particular, an MDDCT strategy based on Gag, Pol, and Nef antigens, was associated with a post-therapy decrease of the viral load set point of one order of magnitude in a subset of patients [11]. However, in this case, and all other MDDCT studies reported so far, post-therapy viral load control was not comparable to that exerted by ART. Although this and other results have suggested that DC-based MDDCTs, if improved, might become an essential therapeutic tool, the correlates of immune control of viral load in the MDDCT responder subpopulation have remained unclear. One possible limitation of the approaches tested so far is using a standardized MDDCT design, administered to all patients independently of their HLA genotype. Given the degree of HLA polymorphism worldwide [23], individual variability can significantly impact immune recognition.

Another potential limitation in the design of the MDDCTs attempted so far is the choice of antigens used for immunization. Indeed, correlation studies between cell-mediated immunity and HIV-1 viral load control suggest that a narrower selection of viral proteins as MDDCT targets might be preferable. In particular, the viral capsid Gag protein represents an attractive target since anti-Gag cell-mediated immunity was repeatedly highlighted as the main immunological correlate of reduced viral load and decreased disease progression [24–29]. Additional evidence suggests that latently HIV-1 infected cells, which are the main obstacle to the cure of HIV-1, express low levels of the Gag protein and could thus be detected and eventually eliminated by Gag-specific cytotoxic T-lymphocytes [30, 31]. The particular efficacy of anti-Gag immunity is likely determined by the structural role of the main maturation product of Gag, i.e., the viral core protein p24, which is the building block of the HIV-1 capsid [32]. Several constraints lock p24 within the icosahedral capsid structure, limiting its capability to mutate [33, 34] without, however, abrogating it entirely [35]. Apart from being less effective, cell-mediated immune responses directed against viral targets other than Gag might contribute to immune hyperactivation and exhaustion [36]. Moreover, pre-existing cell-mediated immune responses against different viral antigens might hamper MDDCT efficacy.

For this reason, an appropriate conditioning regimen, erasing some of the previously acquired immunity, could constitute a valuable addition to MDDCT. In this regard, lymphorestrictive approaches in the setting of chronic diseases characterized by malignant immune hyperactivation, such as HIV/AIDS or cancer, may paradoxically result in enhanced cell-mediated responses against the diseased cells [37, 38]. Of note, none of the MDDCT approaches attempted so far have been associated with any conditioning regimen.

Given these premises, we designed and tested a personalized MDDCT using autologous HIV Gag peptides from patients undergoing long-term suppressive ART to decrease the reservoir of HIV-infected cells. This MDDCT was administered in the context of a larger clinical trial (clinicaltrials.gov ID: NCT02961829) which also examined the effects of a conditioning regimen consisting of auranofin to decrease the central memory T-cell pool encompassing the viral reservoir [39–41]) and nicotinamide, favoring HIV escape from latency to facilitate recognition of the HIV-infected cells [42, 43]. To assess the immunogenicity of our personalized MDDCT strategy and help guide the choice of analytical treatment interruption (ATI) in a larger planned trial, we performed an *interim* analysis of immunologic parameters in the MDDCT recipients.

## Materials and methods

### The general plan of the study

Between February 2015 and May 2016, 10 chronically HIV-1-infected patients were included in treatment arms 5 and 6 of the SPARC-06 Clinical Trial (NCT02961829) to receive an autologous MDDCT without or with a conditioning regimen consisting of nicotinamide and auranofin [39]. We enrolled males ≥ 18 years under suppressive ART with undetectable viral load for more than two years without virologic failure (Table 1). CD4+ T-cell counts at enrollment were above 500 cells/mm^3^, and CD4^+^ T-cell count nadir was above 350 cells/mm^3^. MDDCT was performed in the study candidates after the first 48 weeks of the intervention period in three doses at baseline (time zero), fifteen days after the first dose, and 30 days after the first dose. There were two significant deviations from the protocol (see Results section). All patients had undetectable viral load upon MDDCT administration except for the two protocol violators (P24 and P26, Table 1), which were viremic and not receiving ART during MDDCT administration.

**Table 1:** Demographic characteristics of MDDCT recipients

Patient ID	Age (years)	Time since diagnosis (years)	Nadir CD4^+^ T-cell count (cells/mm^3^)	Screening CD4^+^ T-cell count (cells/mm^3^)	Pre-ART viral load (copies/mL)	ART regimen		Locus A		Locus B		Locus C		Locus DRB1	
						Current	Intervention	MHC Class I						MHC Class II	
P21	31	10	749	–	98000	TDF + 3TC + FPV + RTV	Dolutegravir + DC	**02**:AJEBB	**03**:AJEYV	**18**:AEDBZ	**40**:AEDCG	**03**:AJFXY	**03**:AJFZD	**07**:ANCXR	**13**:AKKUB
P22	54	7	552	1404	364000	AZT + 3TC + EFV	Dolutegravir + DC	**01**: AJEVP	**03**:AJEZG	**35**:AGNUR	**44**:AGKXV	**04**:AJTUU	**16**:AJSFG	**04**:VVSK	**08**:AKKFM
P23	26	5	490	693	527795	TDF + 3TC + ATV + RTV	Dolutegravir + DC	**01**: AJHDX	**33**:AJHPP	**37**:AGKTW	**58**:AGMCF	**03**:AJSBD	**06**:AJWYX	**04**:AEEWN	**07**:ANBTH
P24	55	22	161	874	480000	TDF + 3TC + EFV	Dolutegravir + DC	**01**: AJEVS	**24**:AJFBS	**40**:AGHBM	**51**:AGHCW	**02**:AJRUK	**15**:AJRUU	**04**:VVSK	**13**:AGKBM
P25	43	21	400	1591	102000	TDF + 3TC + LPV + RTV	Dolutegravir + DC	**02**:AJEXK	**24**:AJFBM	**14**:AGRAG	**51**:ZUDX	**02**:AJRUK	**15**:AJRUU	**01**:ADXMM	**08**:AGFNT
P26	38	6	530	770	12859	AZT + 3TC + EFV	Dolutegravir + Auranofin + Nicotinamide + DC	**24**:AJSFV	**02**:AEDPZ	**15**:AGRAS	**57**:BNK	**02**:AJGWH	**03**:AJGWT	**04**:ANEGN	**16**:ANBUK
P27	24	3	766	839	172029	TDF + 3TC + EFV	Dolutegravir + Auranofin + Nicotinamide + DC	**02**:AJEAZ	**02**:AJEAZ	**15**:AGKPJ	**51**:AGKZJ	**04**:AJFYM	**14**:AGTAE	**11**:ANTEX	**11**:AGMJS
P28	44	6	685	1265	57800	TDF + 3TC + EFV	Dolutegravir + Auranofin + Nicotinamide + DC	**23**:AHPYX	**26**:AJFCW	**14**:ADJFT	**44**:AGRGS	**04**:AJKCT	**08**:AJKEG	**07**:ANCXN	**07**:ANCXN
P29	49	8	505	677	3300	TDF + 3TC + EFV	Dolutegravir + Auranofin + Nicotinamide + DC	**02**:AJEBB	**03**:AJEYV	**07**:AGKMM	**13**:AGKNX	**06**:AJKDJ	**07**:AJKEA	**03**:ANCWU	**10**:VDG
P30	60	13	460	1001	278000	TDF + 3TC + EFV	Dolutegravir + Auranofin + Nicotinamide + DC	**02**:AJEAZ	**02**:AJEAZ	**07**:AGKMF	**51**:AGMAB	**07**:AJHZC	**16**:AJJAB	**15**:ANBUC	**16**:ANBUG

HLA genotypes are shown in bold, followed by the National Marrow Donor Program allele codes

To avoid any effect of auranofin and nicotinamide on MDDC function, auranofin was interrupted 24 weeks before and nicotinamide immediately before the MDDCT.

### MDDCT design

We designed personalized peptides for pulsing of MDDCs, following a multi-step approach for each study candidate. As a first step, each patient’s HIV-1 *gag* sequence was characterized from peripheral blood mononuclear cell (PBMCs) DNA through the generation of genomic sequences after clonal amplification of viral strains through Next Generation Sequencing (NGS) and single genome amplification. For single genome amplification, at least ten clones were generated per patient, as previously described [44]. In the second step, the HIV DNA sequences were translated to amino acid sequences. Briefly, the three frames of translation were aligned to the start of Gag in the HXB-2 reference sequence to determine the correct reading frame using Clustal-Omega [45]. The polypeptides obtained were edited by manual correction, i.e. tryptophans were used to replace spurious stop codons in the middle of the sequence. Clustal-Omega was also used to create a consensus sequence. Additional alignments were then made using the published aligned sequences to map the highly conserved regions of the consensus Gag sequences from each study subject.

In parallel, HLA haplotypes of the same individuals were sequenced. HLA A, B, C, and DR typing was done by sequence-specific oligonucleotide methodology using a LUMINEX 200 apparatus. Briefly, DNA was extracted from the buffy coat of EDTA- anticoagulated peripheral blood with the Qiamp DNA mini kit (Qiagen) and subjected to One lambda LAbtype SSO (Thermo Fisher) according to the manufacturers’ instructions.

Finally, the selection of HIV-1 Gag epitopes was performed by designing peptides from the autologous HIV *gag* sequence and selecting those predicted to be recognized by each individual’s MHC Class I and Class II. The epitopes were predicted using the NetMHCpan v4.0 server [46], which displays an overall accumulated prediction ranking score of 49–78 [47]), as calculated between 2018 and 2021 by The Immune Epitope Database (IEDB) [48]). A number of peptides were selected in positions encompassing amino acids 256–377 of the Gag polypeptide. Those peptides containing more than one cysteine were discarded wherever possible to avoid internal disulfide bonds. The number of peptides designed for each candidate depended on the patients’ affinity for multiple HLA epitopes. Therefore, the higher the number of HLAs predicted to be bound by each peptide, the lower the number of Gag peptides for each candidate. Also, the number of peptides was chosen to keep the immunizing stimuli to a minimum. For example, peptides predicted to bind to multiple HLA haplotypes were preferred over those recognized by a single epitope: to minimize the synthesis costs and to ensure the maximum level of sequence conservation, preference was given to those peptides recognized by the haplotypes of more than one individual. For individuals for whom at least two peptides theoretically binding with high-affinity to more than one of their haplotypes were not found, a higher number of peptides, including peptides outside those above mentioned highly conserved regions, were designed, in order to increase the likelihood of immune recognition. In this manner, we designed 2 to 6 peptides (9-mers) per candidate, as shown in Additional file 11: Table S1. After MDDCT administration, NetMHCpan predictions were reanalyzed using the newly developed Custommune software [49]), which is validated based on documented biological data from the Los Alamos database [50]). The results were used to stratify patients’ ex-post based on the concordance between software predictions [51].

### Peptide synthesis

An automatic desktop synthesizer (PSSM 8 of Shimadzu) was used for the simultaneous solid-phase synthesis of all peptides using the Fmoc procedure. The final peptides were “unprotected” in TFA and purified by HPLC semi-preparation using an Econosil C-18 column (10 μm, 22.5 × 250 mm) and a system of two solvents: (A) trifluoroacetic acid (TFA)/H_2_O (1: 1000) and (B) TFA/acetonitrile (ACN)/H_2_O (1: 900: 100). The column was eluted at a flow rate of 8 mL/min with a gradient of 0 to 80% of solvent B for 45 min. The HPLC analysis was done using a binary HPLC system made by Shimadzu with a UV–vis detector SPD-10AV (Shimadzu), coupled to an Ultrasphere C-18 column (5 μm, 4.6 × 150 mm) that was eluted with system solvents A1 (TFA/H_2_O, 1: 1000) and B1 (ACN/H_2_O/TFA, 900: 100: 1) at a flow rate of 1.0 mL/min and a gradient of 10–80% of B1 for 10 min. The eluates of the HPLC columns were monitored for their absorbance at 220 nm. The molecular weight and the purity of the synthesized proteins were verified by electron spray (LC/MS-2010 Shimadzu). The number of peptides was determined by analyzing the amino acids (Shimadzu).

### MDDC production

The collection of MDDC precursor cells was carried out by leukapheresis with the cell separator Terumo Cobe Spectra at the Blood Center of São Paulo, São Paulo, Brazil. The total blood volume was calculated for each participant, and 1.5 total blood volumes were processed, in peripheral venous access, with continuous flow, at a speed of 50–60 mL/min. After the end of the collection, the leukapheresis product was sent to the Retrovirology Laboratory of the Federal University of Sao Paulo for the separation of monocytes and subsequent differentiation into DCs. Mononuclear cells from leukapheresis products were separated by Ficoll-Hypaque Premium (GE Healthcare® BioSciences, PA, USA) density gradient centrifugation and were cryopreserved in aliquots of 5 × 10^7^ cells/mL in liquid nitrogen using fetal bovine serum (FBS; Gibco Life Technologies®, CA, USA) with 10% dimethyl sulfoxide (DMSO; Merck, Darmstadt, HE, DEU) until further assays were performed. Before use, cells were thawed at 37 °C in a water bath and seeded at 5 × 10^6^/mL in 175 cm^2^ tissue culture flasks (Corning®—Merck, Darmstadt, HE, DEU) in RPMI 1640 medium (Gibco Life Technologies) for two h at 37 °C in a 5% CO_2_ incubator to obtain adherence-isolated monocytes. After incubation, non-adherent cells were removed by washing, and the remaining cells (predominantly monocytes) were differentiated in MDDCs. To this aim, we compared two different procedures to select and optimize the most efficient protocol. In particular, we compared a protocol using IL-4 initially and GM-CSF followed by TNF, IL-1β, and IL-6 (henceforth, IL-4 protocol; Additional file 1: Fig. S1A) with a protocol using IFN-α initially and GM-CSF, followed by LPS (henceforth, IFN-α protocol; Additional file 1: Fig. S1B). Briefly, in the IL-4 protocol, adherent cells were cultured in AIM-V medium (Therapeutic Grade – Gibco Life Technologies) in the presence of 50 ng/ml recombinant human Granulocyte–macrophage colony-stimulating factor (GM-CSF; Cell-Genix®, NH, USA) and 50 ng/ml recombinant human IL-4 (Cell-GenixR, NH, USA) for five days, so as to obtain immature MDDCs (iMDDCs). On day 5, iMDDCs were pulsed with a pool of personalized HIV peptides that was added to the cells (0.2 µg/mL each peptide) overnight. Later, the cells were washed to remove unbound peptide particles and were cultured for an additional two days in an AIM-V medium supplemented with the maturation cytokines IL-6 (100 ng/ml), IL-1β (10 ng/ml), and TNF (50 ng/ml), plus GM-CSF (50 ng/ml) and IL-4 (50 ng/ml; all from Cell-Genix), to obtain mature MDDCs (mMDDCs) pulsed with autologous peptides (Additional file 1: Fig. S1A).

In the IFN-α protocol, adherent cells were cultured in AIM-V medium to which GM-CSF and 500 IU/mL IFN-α-2b (Miltenyi Biotec, Auburn, CA, USA) were added on days 0 and 1 to obtain iMDDCs. On day 2, a pool of personalized HIV peptides was added to the cells (0.2 µg/mL each peptide) overnight. The following day, six h before cell harvest, maturation of iMDDCs was induced by adding 5 IU/mL lipopolysaccharide (LPS-SM, Ultrapure InvivoGen, San Diego, CA, USA). After the incubation period, mMDDCs were recovered on ice and washed three times with sodium chloride solution (0.9% NaCl, USP grade; Hospira, Lake Forest, IL) (Additional file 1: Fig. S1B).

Before reinfusion in patients, both iMDDCs and the final MDDCT were subjected to quality controls and immunophenotyping by flow cytometry analysis. For validation tests of DC maturation protocols, cell mortality was assessed using the cell viability kit (BD Biosciences) according to the manufacturer’s recommendations. For assessing the viability of MDDCs before MDDCT administration, the amine-reactive fixable LIVE/DEAD stain (Gibco Life Technologies, OR, USA) was used, by staining for 30 min at 4 °C. For further staining, after thorough washing with phosphate-buffered saline (PBS), cells were incubated for 20 min at 4 °C and assayed using the following monoclonal antibody panel: CD11c APC or V450 (clone B-ly6), CD14 Pacific Blue (clone M5E2), HLA-DR FITC (clone G46–6), CD80 PE (clone L307.4), CD86 FITC (clone 2331 FUN-1) and CCR7 PerCP (clone 150,503). All the mAbs were obtained from BD Biosciences®, CA, USA, except CCR7 (R&D Systems®, MN, USA). Subsequently, cells were washed with FACS buffer (0.2% albumin and 0.1% sodium azide in PBS) and then fixed with 2% paraformaldehyde in PBS. Data were acquired on an LSR Fortessa Flow Cytometer (BD Biosciences) using the DIVA software, and the analysis was performed with FlowJo vX 0.7 software (Tree Star®, OR, USA).

### MDDCT immunogenicity analysis

The effect of MDDCT on specific anti-HIV immune response in treated patients was assessed in patients’ PBMCs collected from total blood on days 0, 15, 30 (i.e.*,* upon each dose of therapeutic immunization), and 120 (i.e.*,* post-immunization follow-up). Total blood was drawn before each MDDCT dose to avoid confounding effects of MDDCT on each baseline. For this purpose, cells were plated at a concentration of 1 × 10^6^/mL in 96-well round-bottomed plates in 200 μl of RPMI 1640 medium plus 10% FBS. Cells were either left unstimulated or incubated with 1 μg/mL of personalized peptides for 48 h in an incubator at 37 °C and 5% CO_2_. Six hours before cell harvest, the positive control wells (C^+^) were stimulated with 1 μg/mL staphylococcal enterotoxin B (SEB, Merck OR, USA). After one h stimulation with SEB, 20 μg/mL of Brefeldin A (BFA, Merck) was added to the plate to block any further protein transport. Background controls (C^−^) consisted of PBMCs cultured in the absence of peptides and were used as blank, i.e., subtracted from the percentage of cytokine-producing lymphocytes obtained from wells incubated with the peptides. To evaluate immune responses/activation, PBMCs were first fixed and permeabilized using the Cytofix/Cytoperm and Perm/Wash kits (BD Biosciences CA, USA), following the manufacturer’s recommendations. Cells were then incubated with a fixable live/dead stain (Gibco Life Technologies OR, USA) to check their viability and stained with a panel of antibodies including α-CD3 (V450; clone UCHT1), α-CD4 (BV605; clone RPA-T4), and α-CD8 (APC-H7; clone SK1) as well as antibodies against the intracellular cytokines IFN-γ (PerCP-Cy5.5; clone B27), TNF (PE-Cy7; clone MAb11), and IL-2 (FITC; clone MQ1–17H12) (all from BD Biosciences CA, USA). Data acquisition was performed on an LSR Fortessa Flow Cytometer, using the DIVA software, and the analysis was performed with FlowJo vX 0.7. All samples and controls were analyzed in triplicate.

### IFN-γ measurement in serum

Patient serum samples were collected upon administration of the first MDDCT dose (day 0), upon dose 2 (day 15), and dose three administration (day 30). The level of IFN-γ was measured using an ELISA technique following the guidelines provided by the manufacturer of the kit (BD OptEIA™ Human IFN-γ ELISA Set—BD Biosciences®).

### Proviral DNA quantitation in rectal biopsies

Viral DNA was measured to estimate the viral reservoir by qPCR as previously described [52–54].

### Data analysis

Comparisons between two groups were conducted by chi-square testing, relative risk analysis and non-parametric Wilcoxon test. Comparisons between more than two groups were conducted by one-way ANOVA, followed by Dunnet’s post-test, or by two-way ANOVA followed by Tukey’s post-test. When appropriate, a *logit* transformation was applied to the data to restore normality before the statistical test. To allow for paired statistical analysis of the differences, patients with a missing value in a time point were excluded from that specific statistical analysis, but all available values were included when plotting the overall data. All analyses were conducted using the Prism v.6 software (GraphPad® Software Inc, CA, USA).

## Results

### Optimization of MDDCT production

To optimize the preparation of MDDCs, we initially tested two different protocols (i.e., IL-4 based and IFN-α based) in terms of differentiation and maturation (Additional file 1: Fig. S1). The protocol based on IFN-α proved more effective when differentiating MDDCs from frozen PBMCs, leading to higher cell viability (Additional file 2: Fig. S2). Moreover, when comparing the phenotypic differentiation profile of DCs, the protocol based on IFN-α was associated with higher expression of the costimulatory molecule CD80 (Additional file 3: Fig. S3). In light of this evidence, the IFN-α-based protocol was adopted for the present study. Finally, we compared the viability of DCs differentiated after thawing of frozen PBMCs to that of DCs frozen after isolation. Despite its potential advantages in streamlining the protocol, the use of frozen DCs was associated with lower viability and deemed suboptimal for further use (data not shown).

Overall, these results support the differentiation of MDDCs based on FBS-frozen PBMCs and the use of IFN-α as the optimal protocol for our MDDCT production.

### Phenotypic analysis of MDDCs used for MDDCT

**Fig. 1:**
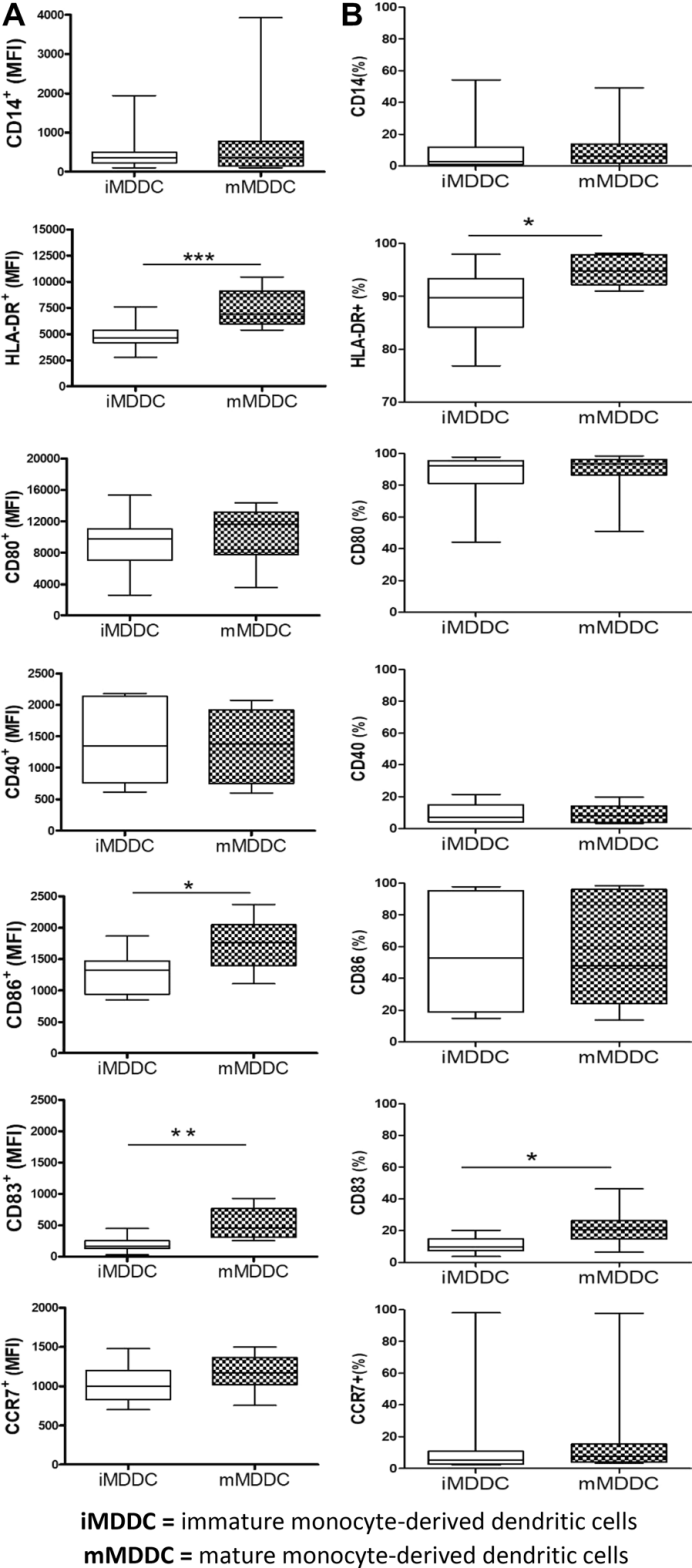
Phenotypic characterization of immature and mature MDDCs used for MDDCT. Panels A,B) PBMCs were isolated from the total blood of the enrolled individuals and induced to MDDC differentiation/maturation as depicted in Additional file 1: Fig. S1. Both immature MDDCs (iMDDC) and mature MDDCs (mMDDCs) were analyzed by flow cytometry for the expression of the activation/maturation markers HLA-DR, CD80, CD86, CD83, CD40, and CCR7 according to the gating strategy depicted in Additional file 1: Fig. S7A. Box plots and whiskers represent the median fluorescence intensity of each marker (**A**) or the percentage of cells expressing each marker (**B**). Data are expressed as median ± min/max and were analyzed by the non-parametric Wilcoxon test (N of patients = 10).** p < 0.01

**Fig. 2:**
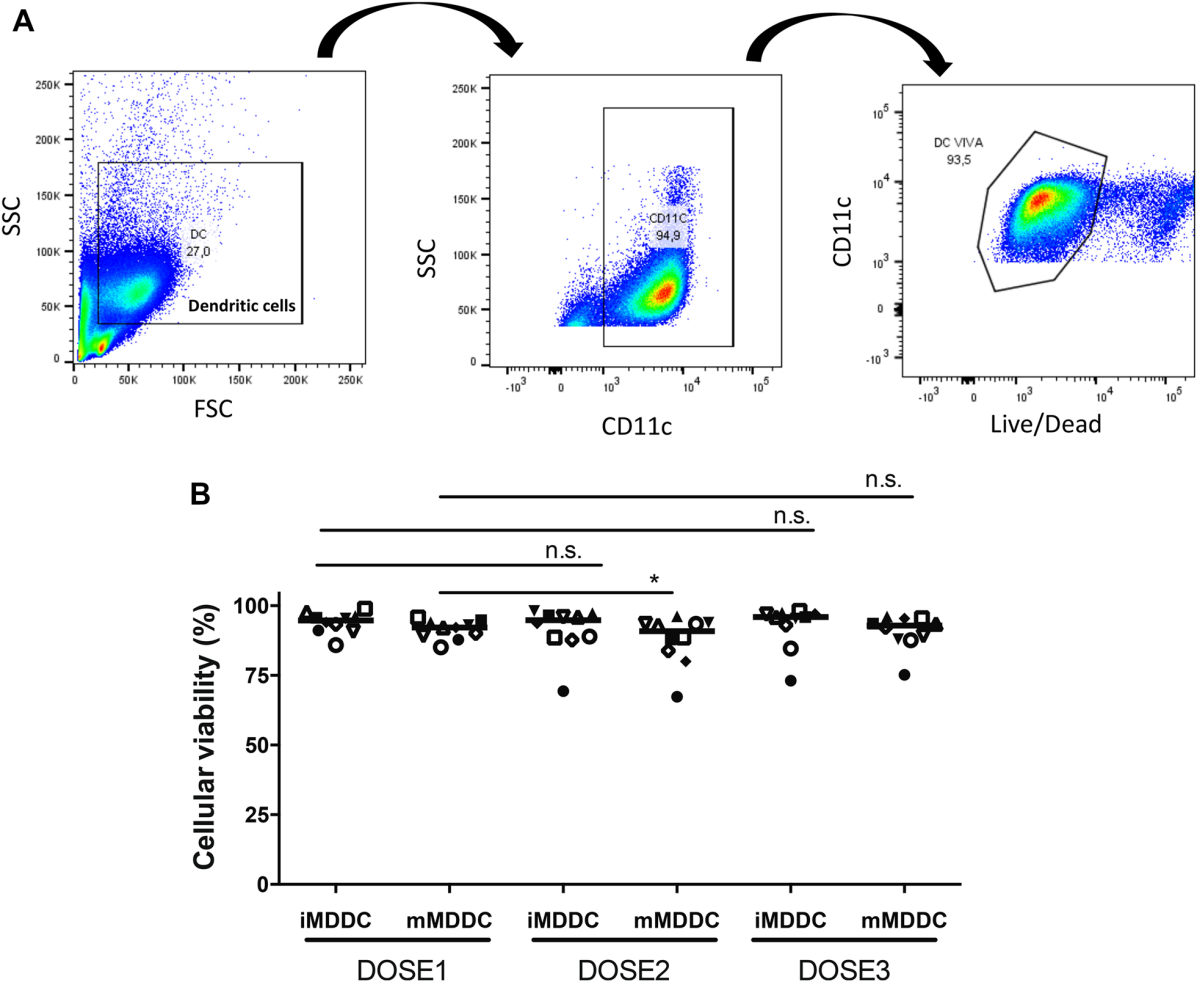
Viability of MDDCs used for each MDDCT dose preparation. Viability was assessed by flow cytometry following staining with a LIVE/DEAD fixable stain according to the gating strategy shown in (**A**). The horizontal line in the graph of panel **B** indicates the median (N of patients for each dose = 10). Data were analyzed by Two-Way ANOVA. * p < 0.05

We then examined by flow cytometry the phenotypic characteristics of MDDCs used for MDDCT administration (Fig. 1). The characteristics of the enrolled patients are detailed in Table 1. The gating strategy used for the analysis is depicted in Additional file 4: Fig. S4. The induction of maturation, a prerequisite of MDDCT administration (Additional file 1: Fig. S1), was associated, as expected, with increased expression of the activation/maturation markers HLA-DR, CD80, CD86, and CCR7 (Fig. 1). While the expression of CD80 and HLA-DR marks DC activation, but can also be upregulated by tolerogenic DCs, the expression CD83 and CD86 is a marker of mature DCs, supporting the effect of the protocol in specifically inducing DC maturation [55]. Moreover, the viability of MDDCs used for MDDCT was similar throughout the three doses administered (median viability > 90% for all doses) except for a small, albeit significant decrease observed in MMDDCs administered during the second dose (Fig. 2A, B).

### Administration of peptide-pulsed MDCCs is associated with T-cell responses

**Fig. 3:**
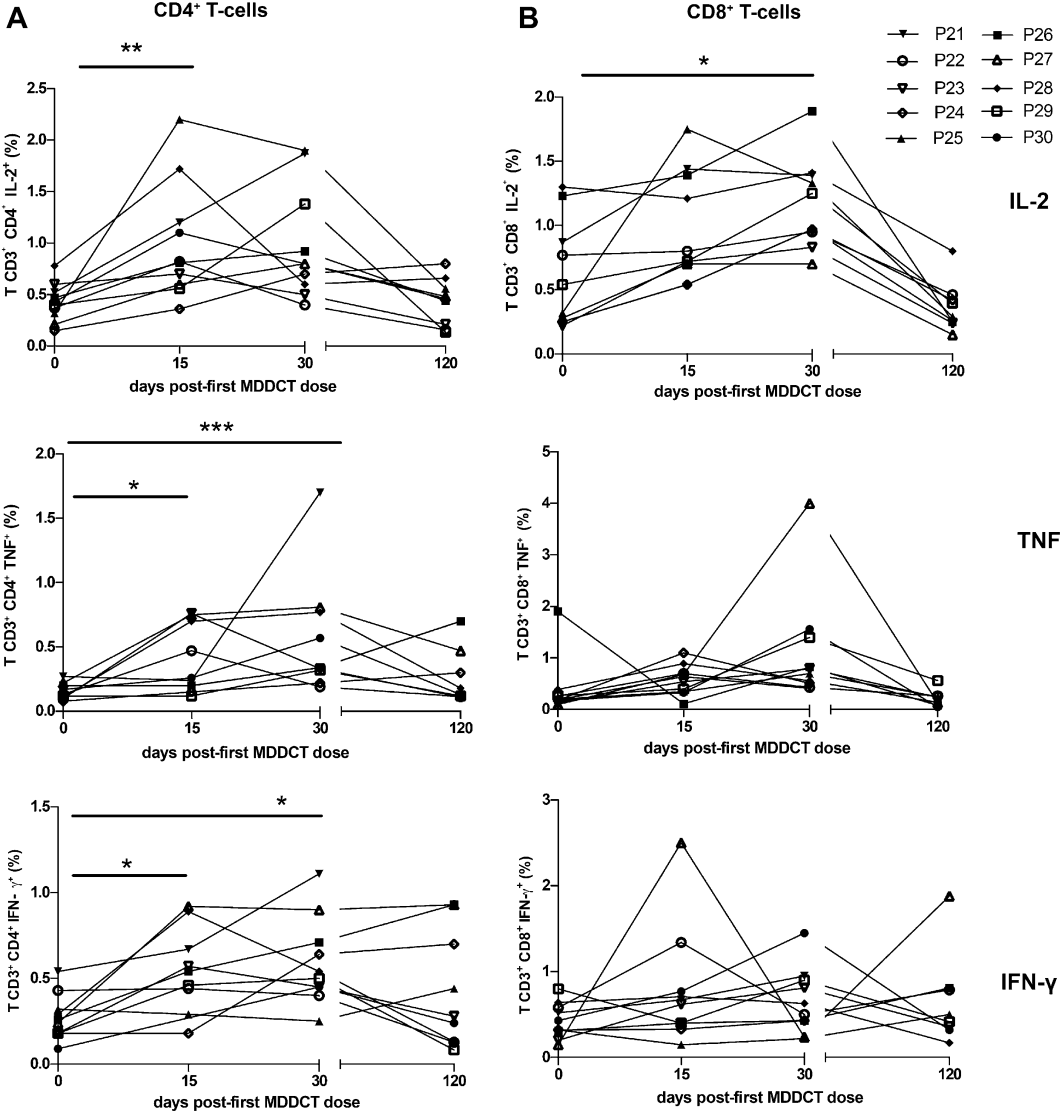
Immunogenicity of MDDCT. Ten recipients (Table 1) received three doses of the personalized MDDCT. PBMCs were collected upon administration of the first MDDCT dose (day 0), upon dose 2 (day 15), and dose 3 administration (day 30), as well as during the post-therapy follow-up (day 120). Isolated PBMCs were stimulated in vitro with the autologous Gag peptides that were used for MDDCT. Production of IFN-γ, IL-2, and TNF by CD4^+^ and CD8^+^ T-lymphocytes was evaluated by flow cytometry. After applying the *logit* transformation to restore normality, data of patients for whom all time points were available for a given cytokine were analyzed by one-way ANOVA followed by Dunnet’s post-test. *p < 0.05; **p < 0.01; ***p < 0.001

Each dose of the autologous, personalized MDDCT was then administered to 10 chronically HIV-1-infected individuals. A total of 10^7^ cells were administered per infusion. No adverse effects were observed upon vaccine administration. To evaluate the immunogenicity of MDDCT, we examined by flow cytometry the peptide-specific functional T-cell responses in vitro, following the gating strategy depicted in Additional file 5: Fig. S5. The results showed increased production, in both CD4^+^ and CD8^+^ T lymphocytes, of IFN-γ, IL-2, and TNF. This upregulated cytokine production peaked upon administration of dose 3 of the MDDCT and was then reverted during the post-therapy follow-up (Fig. 3). Of note, this increase was not observed in matched positive control samples after stimulation with SEB or in unstimulated samples. (Additional files 6, 7: Fig.s S6, S7), thus supporting a specific effect of the MDDCT in inducing response to the peptide stimulus. We also performed qualitative analyses evaluating the number of study volunteers who responded to the MDDCT, comparing the cytokine levels at baseline (day zero) to the cytokine increment on day 15, day 30, and day 120. We chose an arbitrary increase of at least 50% in the cytokine levels upon MDDCT from baseline to define an MDDCT response and compared the results of *in-vitro* peptide-stimulated and unstimulated cells (Table 2). The analysis showed a significant increase in cytokine production in cells stimulated ex vivo with Gag-derived peptides compared to unstimulated cells, suggesting a high proportion of immune responders among individuals subjected to MDDCT (Table 2).

**Table 2:** The proportion of responders among MDDCT recipients at each time point post-MDDCT

Cytokine and comparison	Proportion of post-vaccination responders				Chi-Square (peptide stimulated vs unstimulated)
	Peptide stimulated		Unstimulated		
Day 15 vs day 0	CD4^+^ T-cells	CD8^+^ T-cells	CD4^+^ T-cells	CD8^+^ T-cells	*p* < 0.00001
IL-2	8/10	6/10	3/10	2/10	
TNF	5/10	9/10	5/10	3/10	
IFN-γ	5/9	4/10	0/10	1/10	
Day 30 vs day 0	CD4^+^ T-cells	CD8^+^ T-cells	CD4^+^ T-cells	CD8^+^ T-cells	*p* < 0.00001
IL-2	6/9	7/10	2/9	3/9	
TNF	9/10	8/10	3/10	2/10	
IFN-γ	8/10	4/10	2/10	2/10	
Day 120 vs day 0	CD4^+^ T-cells	CD8^+^ T-cells	CD4^+^ T-cells	CD8^+^ T-cells	*p* < 0.017
IL-2	5/9	1/9	2/9	0/9	
TNF	5/9	2 /9	2/9	3/8	
IFN-γ	5/9	4/9	2/9	2/9	

The data used to calculate responses are shown in Fig. 3 and Additional file 7: Fig. S7. For each condition/cytokine, a responder was defined by a ≥ 50% increase in the percentage of cytokine production as compared to baseline (i.e. day 0). Chi-square analysis was performed using pooled data of all cytokines and comparing the proportion of responders in peptide-stimulated cells vs unstimulated cells of the same individuals

Moreover, the responses in individuals receiving MDDCT only were generally similar to those receiving MDDCT and conditioning regimen, further supporting a specific immunogenic effect of MDDCT (Additional file 8: Fig. S8). The only exception was a significant increase in CD8^++^ TNF^++^ cells among patients receiving MDDCT and condioning regimen at 30 days post-first MDDCT administration. On the other hand, when IFN- γ levels were measured in serum, a trend toward a significant increase over time was observed in those individuals who had received the MDDCT after the auranofin/nicotinamide conditioning regimen (Additional file 9: Fig. S9) notwithstanding the short half-life and stability of this cytokine in serum [56].

**Fig. 4:**
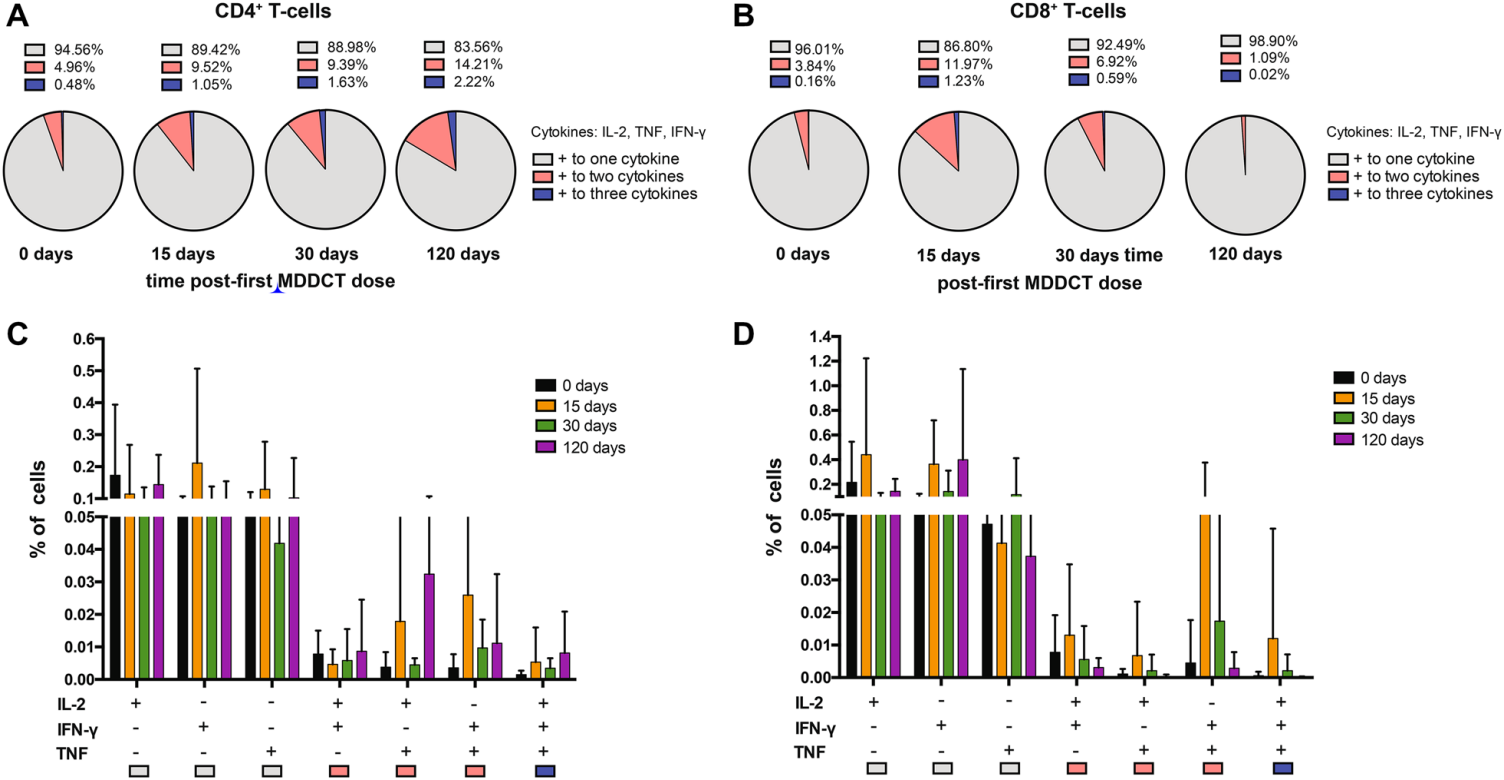
Frequency of polyfunctional CD4^+^ and CD8^+^ T-lymphocytes following MDDCT. PBMCs were collected upon administration of the first MDDCT dose (day 0), upon dose 2 (day 15), and dose 3 administration (day 30), as well as during the post-therapy follow-up (day 120). Isolated PBMCs were stimulated in vitro with the autologous Gag peptides that were used for MDDCT. Production of IFN-γ, IL-2, and TNF by CD4^+^ and CD8^+^ T-lymphocytes was evaluated by flow cytometry. Panels A,B) Frequency of cells expressing one or more immune-mediator cytokine (IL-2, TNF and IFN-γ). Pie charts show the relative percentage, among cells expressing at least one cytokine, of CD4^+^ (**A**) and CD8^+^ (**B**) T-lymphocytes expressing one, two, or three cytokines. Panels **C**, **D**) Bar graphs showing the absolute frequency of CD4^+^ (**C**) and CD8^+^ (**D**) T-lymphocytes expressing each cytokine combination analyzed. Data are expressed as mean ± SD and were analyzed by two-way ANOVA. (N of patients for each dose = 10)

To further characterize the immunogenicity of the MDDCT, we evaluated peptide-specific T-cell polyfunctional responses, which have been previously described as correlates of immune control of the infection [57]. Polyfunctionality was assessed by flow cytometry in the PBMCs obtained after each MDDCT dose, after 48 h of cultivation with autologous peptides, using the gating strategy depicted in Additional file 5: Fig. S5. Our results showed an increase in the frequency of CD4^+^ T-lymphocytes characterized by simultaneous expression of at least two of the three cytokines analyzed (i.e.*,* IFN-γ, IL-2, and TNF) (Fig. 4A). In line with this, within the bulk of the cytokine-producing CD4^+^ T-cells, the proportion expressing only one of the three cytokines decreased significantly from the second to the third MDDCT dose. A similar, although less evident, result was observed when analyzing CD8^+^ T-cells (Fig. 4B). Although the multiple-comparison post-test (Tukey) could not highlight significant differences over time, the two-way ANOVA test adopted did recognize the positivity to one- or multiple-cytokines as the main source of variation (P < 0.01). When the types of cytokines produced by the T-cells were analyzed separately, there was a significant increase in TNF^+^ IFN-γ^+^ CD4^+^ T-cells upon dose two compared to baseline (Fig. 4C). Again, this difference was not visible in CD8 + T-cells (Fig. 4D). To explore whether time on suppressive ART might correlate with MDDCT response, we compared cytokine responses between groups of individuals under long-term suppressive ART (i.e. > 10 years of suppressive ART) and individuals under mid/short term suppressive ART (i.e. < 10 years). The results showed comparable responses between the two groups (Additional file 10: Fig. S10).

Taken together, these data indicate that MDDCT can evoke peptide-specific immune responses, potentially increasing T-cell polyfunctionality during the MDDCT period.

### MDDCT with highly conserved Gag peptides is associated with virologic response

We then evaluated the virologic response to MDDCT and its possible correlates in order to estimate the feasibility of analytical treatment interruption (ATI) in MDDCT recipients. To this aim, we performed an *interim* analysis of viral DNA in rectal biopsies (RB) in the individuals subjected to MDDCT. We chose this tissue since DNA in RB strongly correlates with immune-mediated control of viral load in the absence of ART [58]. The results showed that two subjects, P27 and P29 (Table 1), displayed undetectable viral DNA in RB [59]. Of note, both subjects had detectable viral DNA in RB at baseline, supporting an effect of the treatment in reducing viral burden. Both individuals with undetectable HIV DNA belonged to the group that had received both the conditioning regimen and the MDDCT (Table 1). However, the difference in the proportion of individuals displaying undetectable viral DNA was not significant between the two groups of MDDCT recipients [Groups 5 and 6 (P = 0.4286, intention-to-treat analysis; P = 0.4444, per-protocol analysis; Fisher’s exact test)] [59]. These analyses (both intention-to-treat and per-protocol analysis) were performed to rule out the contribution, to the final result, of two protocol violators (P24 and P26), who, before MDDCT administration, had suspended ART unbeknown to the study investigators.

**Fig. 5:**
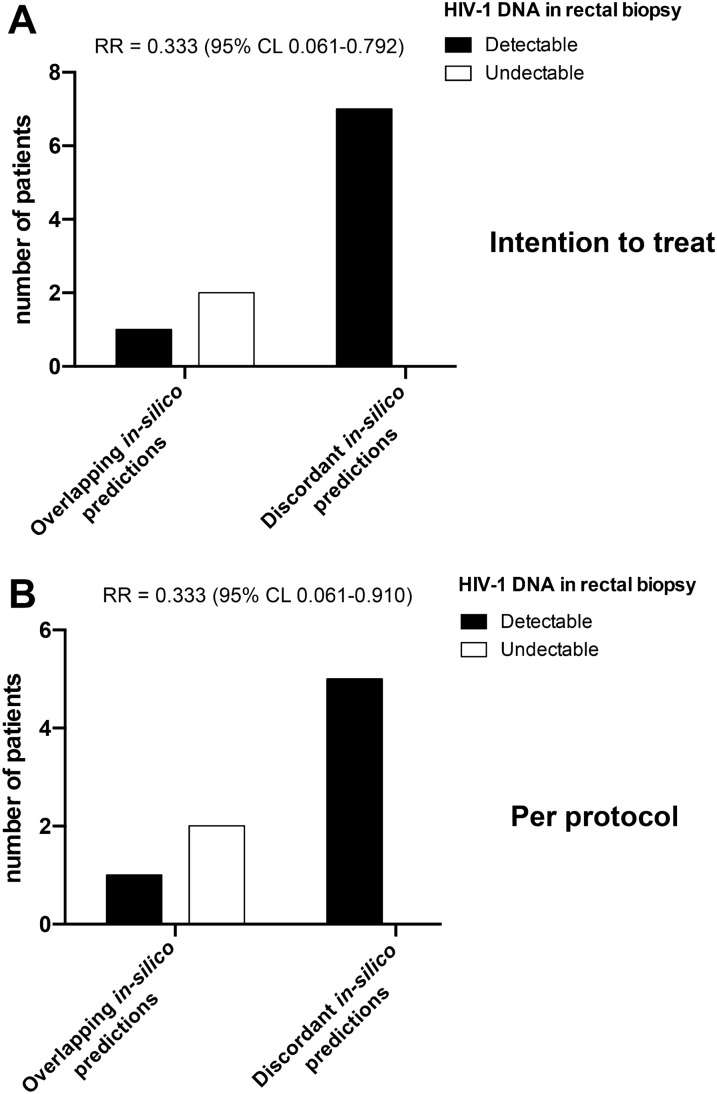
The proportion of patients displaying an undetectable HIV-1 DNA level in rectal biopsies at the end of all treatments. Panels A,B) MDDCT recipients were subjected to rectal biopsy before all investigational interventions (baseline) and at the end of all treatments. Patients were stratified based on the concordance (or lack thereof, i.e. concordance for 50% or less of the epitopes administered) of predicted epitopes between the online tools NetMHCpan (http://www.cbs.dtu.dk/services/NetMHCpan/) and Custommune (www.custommune.com). The relative risk (RR) is referred to the risk of having detectable viral DNA as calculated by intention-to-treat (**A**) and per-treatment (**B**) analysis

To understand the correlates of viral DNA abatement, we then analyzed the accuracy of the predictions of NetMHCpan using a newly developed software (Custommune: www.custommune.com; [51]). This software is based on peptide affinity calculations and selects only those peptides in positions corresponding to epitopes validated by biological data for the matched HLA. Interestingly, the subjects displaying undetectable viral DNA were the only two patients whose entire set of peptides was validated by Custommune against biological data for effective HLA binding (LosAlamos database) (P = 0.0157, intention-to-treat analysis; P = 0.0350, per-protocol analysis; Chi-square) (Fig. 5).

## Discussion

The present study shows the feasibility of using a personalized Gag-based MDDCT to increase the adaptive immune response against HIV in patients undergoing suppressive ART for prolonged periods. In particular, epitopes from highly conserved sequences of the Gag region of HIV could be ideal candidates if they are administered following an appropriate conditioning regimen.

A previous report of post-treatment control of viremia in two macaques infected with the HIV homolog SIVmac251 [60] may help clarify the results of the present study. The macaques had received ART in combination with an experimental treatment with immune-modulating drugs. Although the virus was not eradicated, the macaques showed, upon suspension of all therapies, control of viremia, which was associated with anti-Gag cell-mediated immunity [60]. The most robust immune responses were directed against an amino acid sequence highly conserved in both human and simian lentiviruses (Gag_256-377_; amino acid numbering is according to the HIV Gag epitope map in the Los Alamos HIV database: https://www.hiv.lanl.gov/content/immunology/maps/ctl/Gag.html; accessed 8 June 2019). Also, in the present study, the sequences of the epitopes used to immunize the subjects who eventually displayed an undetectable viral DNA mapped to the highly conserved portion of p24 that is homologous to that identified in the post-treatment controller macaques.

Interestingly, the highly conserved sequence of Gag associated with immune control corresponds to a portion of the protein responsible for multimerization. Furthermore, assembly of p24 displays a recursive pattern with the hexamer being the fundamental unit and hexamers of hexamers being necessary for further assembly of the capsid structure [34]. Thus, the constraints to which this protein portion is subjected may limit the development of viable immune escape mutants.

It is conceivable that the conditioning regimen to which the study subjects had been subjected before MDDCT administration eventually contributed to the result obtained. On the other hand, we were not able to correlate the duration time of suppressive ART with MDDCT function.

It is known that immune control of HIV infection correlates with the quality of the specific response of T-lymphocytes to the antigens [19]. Thus, HIV elite controllers and slow progressors often display HIV-specific CD8^+^ T-lymphocytes capable of producing multiple cytokines and capable of proliferating in response to the antigen. In particular, CD4+ T-lymphocytes express high amounts of IFN-γ and IL-2 in response to HIV peptides [20]. Thus, to measure the immunogenicity of MDDCT, we chose to measure IFN-γ, IL-2, and TNF intracellularly in CD4^+^ and CD8^+^ T-lymphocytes at baseline and after MDDCT dosing. The number of candidates that increased in vitro the cytokine levels in CD4^+^ and CD8^+^ T cells upon stimulation with Gag peptides from baseline to days 15 and from baseline to days 30 and days 120 after MDDCT was significant as compared to Gag unstimulated response, providing evidence for immunogenicity of the intervention. Also, the increment of single-cytokine producing cells occurred consistently when IL-2, TNF, and IFN-γ were measured in CD4^+^ and CD8^+^ T-cells, showing induction of an immunological reaction to the MDDCT adopted in the present study. The confirmation that MDDCT was associated with immune reactivity resides in the fact that cytokine production occurred after MDDCT. At 120 days after MDDCT, there was a trend to a decrease in the levels of intracellular cytokines levels comparable to baseline. The monofunctional analysis of the quantification of interleukins somewhat reinforces the concept that the adaptive immune response to HIV disappears among patients undergoing suppressive ART due to the lack of sustained antigenic presentation, suggesting that the immune reinforcement in these patients was essential for surveillance and elimination of infected cells. In this regard, also the maturation of MDDCs in vitro may have played a role. Thus, the phenotyping and immunogenicity data suggest that the MDDCT adopted in the present study has an immunostimulatory potential to generate an inflammatory cellular response to HIV and can constitute an important additional intervention in strategies aimed at achieving the elimination of the infected cells.

While the correlates of immune protection from HIV-1 progression are not fully understood, polyfunctional immune responses seem to play an important role as suggested by direct evidence on individuals exposed to HIV-1 [61], HIV-1 infected non-progressors [62], and by the association of polyfunctional responses with a lower HIV viral set point [63].

As a weakness in the study’s design, we recognize the absence of an immunogenicity analysis between the application of the last MDDCT dose (day 30) and the sample collected on day 120 which would have allowed better evaluation of the effect last MDDCT dose. Moreover, MDDCs displayed significantly increased markers of maturation but not of CD14, which is an important marker of differentiation of these cells. In addition, although intracellular IL-2 levels may be considered a surrogate marker of T cell proliferative ability [64], we have not been able to explore T cell expansion and degranulation markers in this pilot study to further confirm the generation of MDDCT-related memory immune responses. Although there was a trend towards an increase in simultaneous production of at least two of these cytokines after MDDCT administration in CD4^+^ and CD8^+^ T-lymphocytes, the numerosity of the treatment group did not allow drawing definitive conclusions on this aspect. Therefore, the limited number of study subjects warrants further testing of the present study in a more significant number of individuals.

## Conclusions

This study establishes a novel clinical workflow for personalized MDDCT aimed at targeting HIV-1 reservoirs using a personalized strategy. This strategy combines each individual’s genetic profile and the Gag protein of autologous HIV-1, therefore, providing a proof-of-concept of its potential immunogenicity and efficacy.

## Supplementary Information

**Additional file 1: Fig. S1.** Schematic workflow of the protocols used for the maturation of MDDCs and MDDCT preparation. Maturation of MDDCs from monocytes was induced using either a cytokine cocktail based on IL-4 (longer protocol; Panel A) or a cytokine cocktail based on IFN-α (shorter protocol; Panel B).

**Additional file 2: Fig. S2.** Representative example of the viability of MDDCs under different maturation protocols and conditions. MDDCs were isolated from PBMCs and induced to maturation according to two protocols based on IL-4 and IFN-α as depicted in Additional file 1: Fig. S1. Maturation of MDDCs was performed starting from either freshly collected PBMCs (red bars) or PBMCs frozen in liquid nitrogen upon collection and thawed after 2 weeks. Viability was assessed by flow cytometry using a LIVE/DEAD stain. Data were normalized over the fresh-cell condition.

**Additional file 3: Fig. S3.** Representative example of the maturation profile of MDDCs subjected to alternative differentiation and freezing protocols. MDDCs were isolated from PBMCs and induced to maturation according to two protocols based on IL-4 and IFN-α as depicted in Additional file 1: Fig. S1. Maturation/activation markers CD80 and HLA-DR were analyzed by flow cytometry in cells isolated from fresh PBMCs or from PBMCs thawed after freezing using FBS or a specific freezing medium (as described in the Methods section).

**Additional file 4: Fig. S4.** Gating strategy employed to analyze maturation markers of MDDCs

**Additional file 5: Fig. S5.** Gating strategy employed to analyze cytokine production in T-lymphocytes. Panels A, B. Cytokine production was determined in cells left unstimulated (A) or stimulated ex-vivo with the cell therapy peptides (B).

**Additional file 6: Fig. S6.** Cytokine expression in SEB/brefeldin-stimulated CD4^+^ and CD8^+^ T-lymphocytes following MDDC MDDCT. Data were obtained as described in Fig. 3.

**Additional file 7: Fig. S7.** Cytokine expression in unstimulated CD4^+^ and CD8^+^ T-lymphocytes following MDDC MDDCT. Data were obtained as described in Fig. 3.

**Additional file 8: Fig. S8.** Inter-group comparison of the immunogenicity of MDDCT. Comparison of the levels of IFN-γ, IL-2, and TNF production levels by CD4+ and CD8+ T-cells in individuals receiving MDDCT only (G5) and individuals receiving MDDCT with nicotinamide and auranofin (G6). Data were analyzed by one-way ANOVA followed by Dunn´s post-test. *p < 0.05.

**Additional file 9: Fig. S9.** Impact of MDDCT on the level of IFN- γ in plasma. The Fig. shows the level of IFN-γ in individuals receiving the personalized dendritic-cell therapy (DCT), alone (G5, in black) or following the conditioning regimen consisting of auranofin + nicotinamide (G6, in blue). IFN-gamma was measured by ELISA testing, and data were analyzed by two-way ANOVA. The P-value reported refers to the interaction between time and the previous conditioning regimen.

**Additional file 10: Fig. S10.** Impact of the time on ART on cytokine production post-MDDCT. The graph visualizes the relation between the time in days since the first MDDCT dose and the expression of immune response mediating cytokines in CD4^+^ (Panel A) and CD8^+^ (Panel B) T-cells. Patients were stratified into two categories based on the overall time of ART administration since their diagnosis (i.e. > 10 years, “long” and < 10 years, “mid/short”). The lines on the graph represent linear regression slopes for each group, and the gray areas indicate 95% confidence intervals for each line.

**Additional file 11: Table S1.** List of peptides used for pulsing MDDCs of each individual. Enneamers (9 mers) were designed according to their predicted immunogenicity in a given individual, as described in the main text. Peptides were then used to pulse MDDCs before their reinfusion in MDDCT recipients according to the workflow depicted in Additional file 1: Fig. S1. Note that some autologous peptides are common to more than one patient.

## Data Availability

All data and material will be deposited at GitLab and will be available to readers.
