# Supplementary figures and images for: Immunogenicity of personalized dendritic-cell therapy in HIV-1 infected individuals under suppressive antiretroviral treatment: interim analysis from a phase II clinical trial
Source: AIDS Res Ther. 2022 Jan 12;19:2. doi: 10.1186/s12981-021-00426-z (PMC8753935; doi:10.1186/s12981-021-00426-z)

**A**

### IL-4 protocol for DC preparation

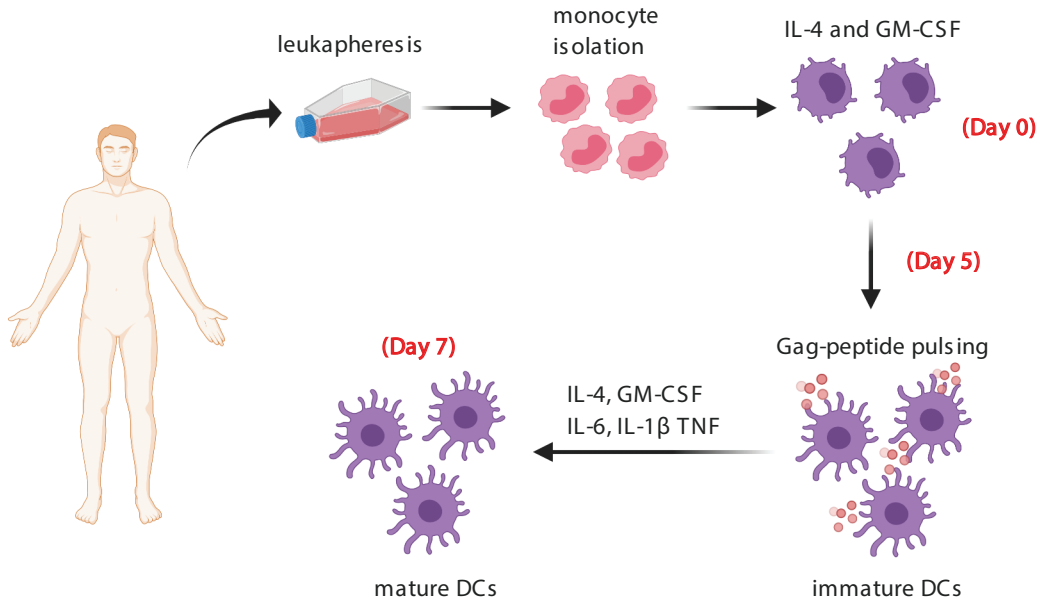**B**

### IFN- $\alpha$ protocol for DC preparation

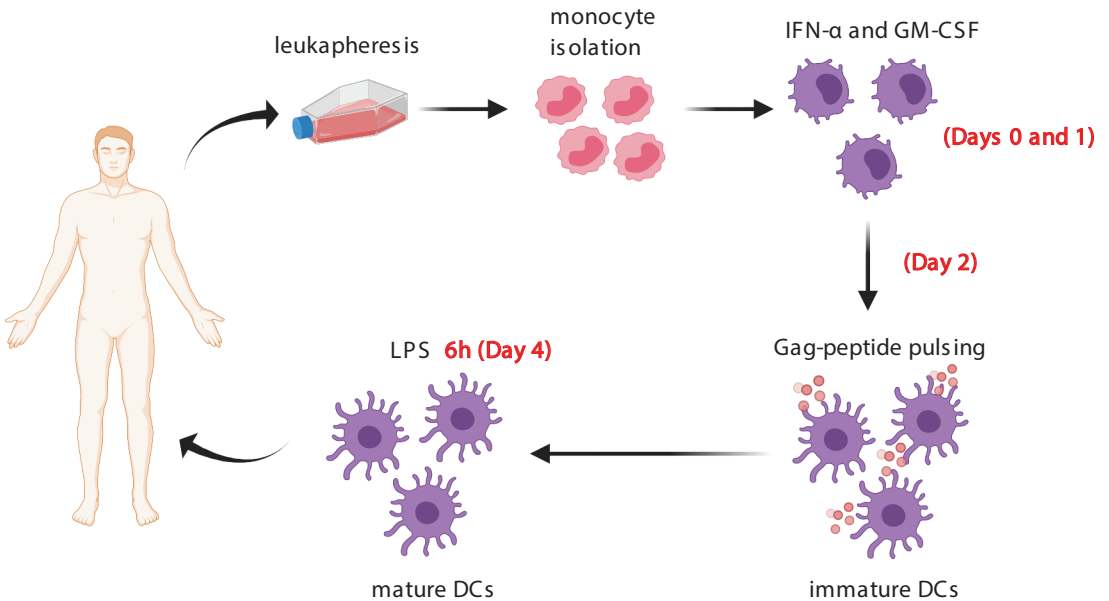

Supplement: Supplementary file 1 — Additional file 1: Fig. S1. Schematic workflow of the protocols used for the maturation of MDDCs and MDDCT preparation. Maturation of MDDCs from monocytes was induced using either a cytokine cocktail based on IL-4 (longer protocol; Panel A) or a cytokine cocktail based on IFN-α (shorter protocol; Panel B). [file 12981_2021_426_MOESM1_ESM.pdf]

**A**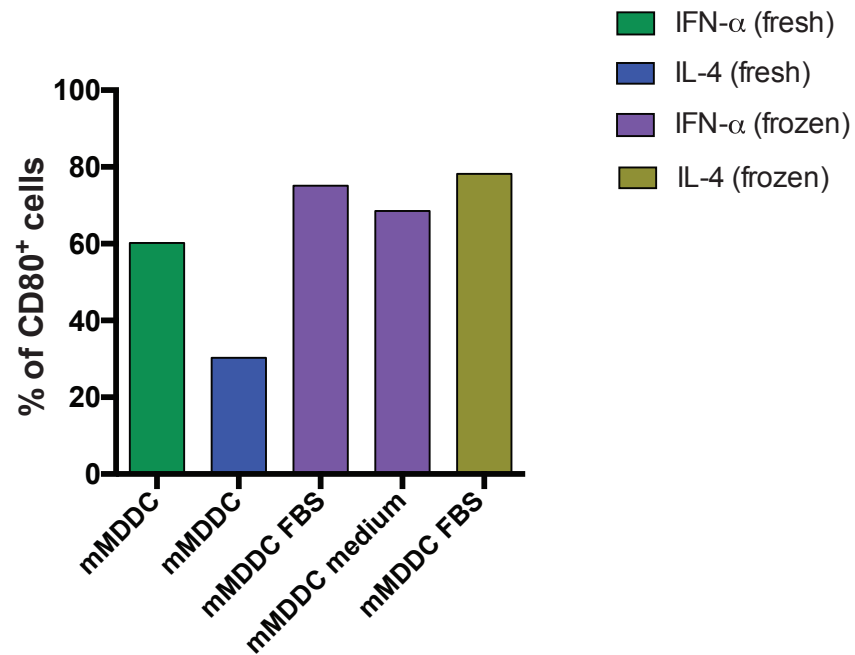**B**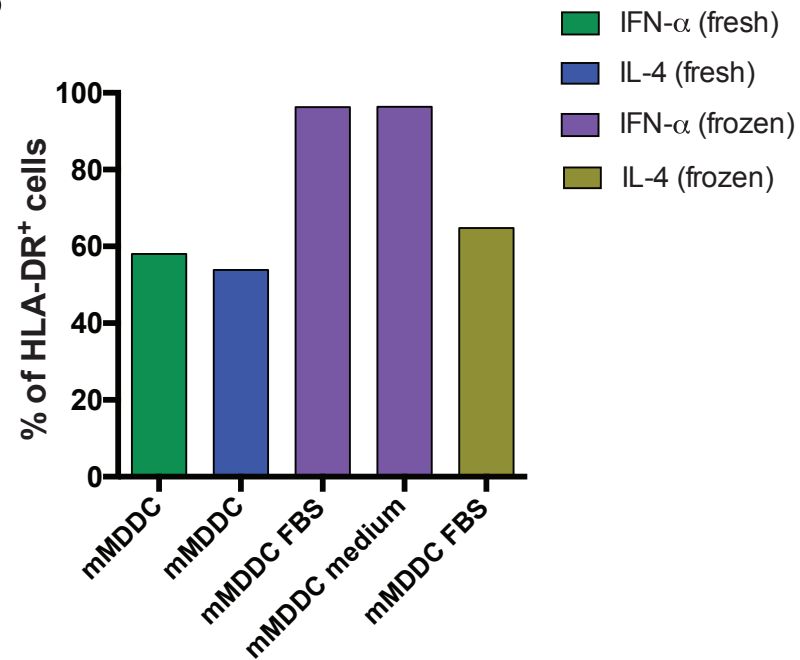

Supplement: Supplementary file 3 — Additional file 3: Fig. S3. Representative example of the maturation profile of MDDCs subjected to alternative differentiation and freezing protocols. MDDCs were isolated from PBMCs and induced to maturation according to two protocols based on IL-4 and IFN-α as depicted in Additional file 1: Fig. S1. Maturation/activation markers CD80 and HLA-DR were analyzed by flow cytometry in cells isolated from fresh PBMCs or from PBMCs thawed after freezing using FBS or a specific freezing medium (as described in the Methods section). [file 12981_2021_426_MOESM3_ESM.pdf]

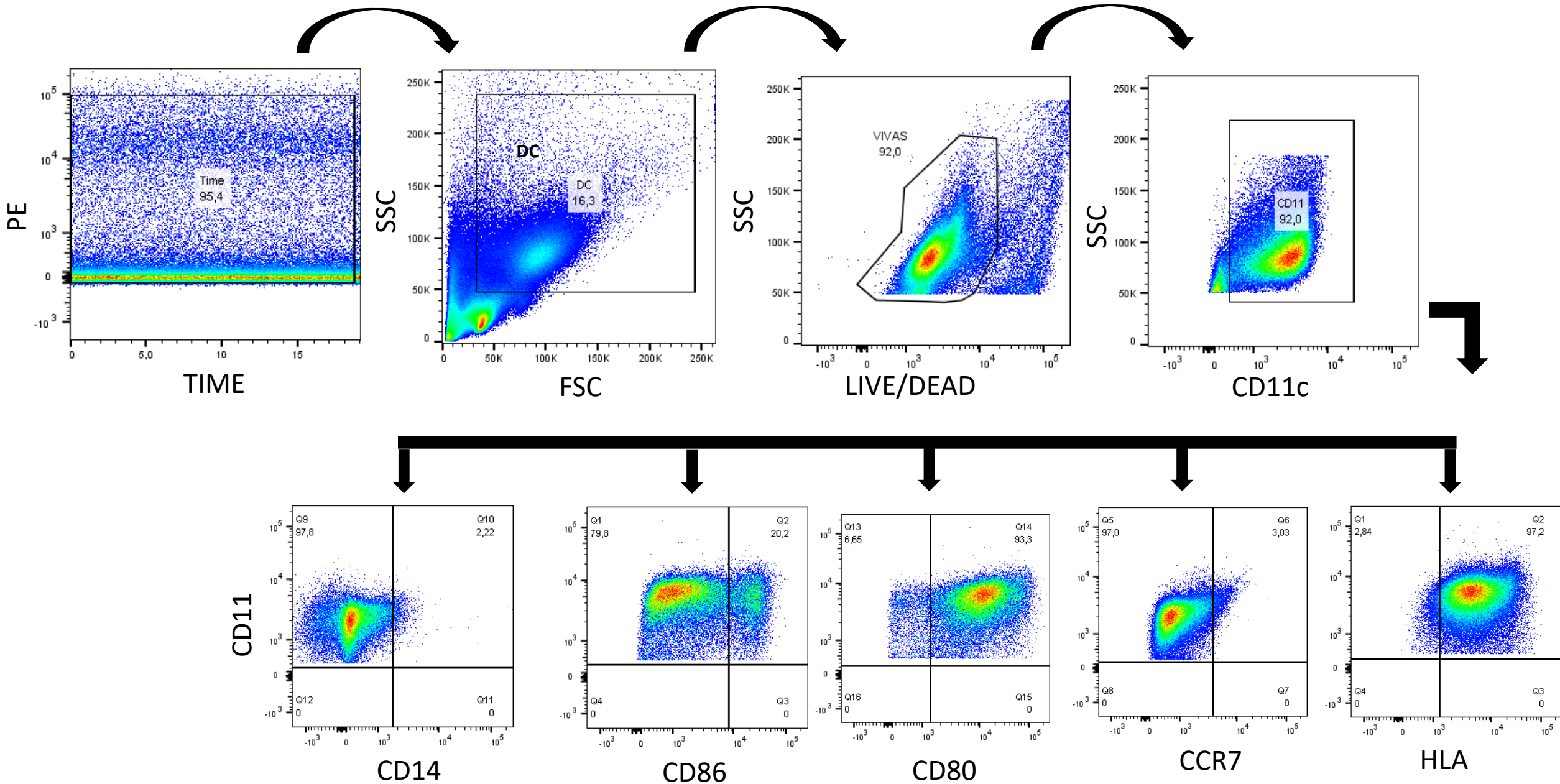

Supplement: Supplementary file 4 — Additional file 4: Fig. S4. Gating strategy employed to analyze maturation markers of MDDCs [file 12981_2021_426_MOESM4_ESM.pdf]

A

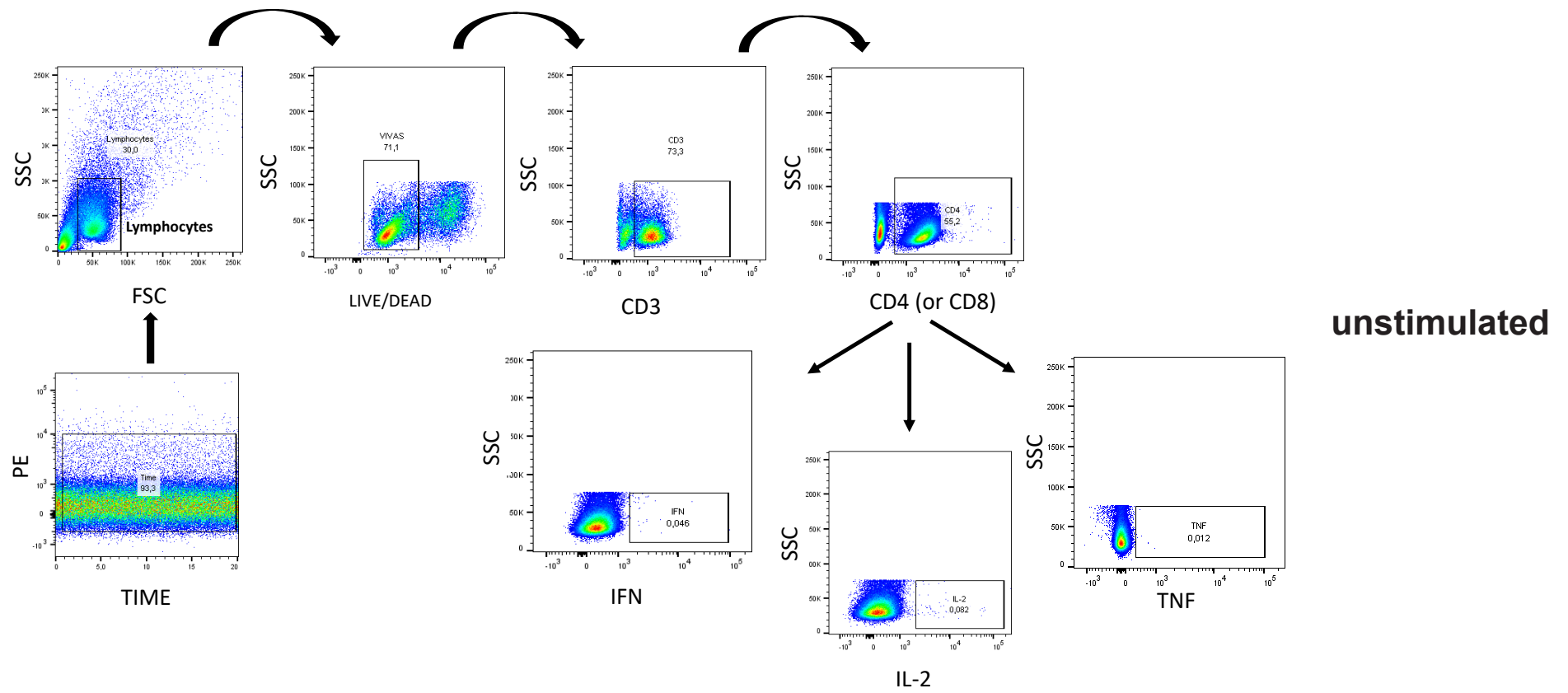

B

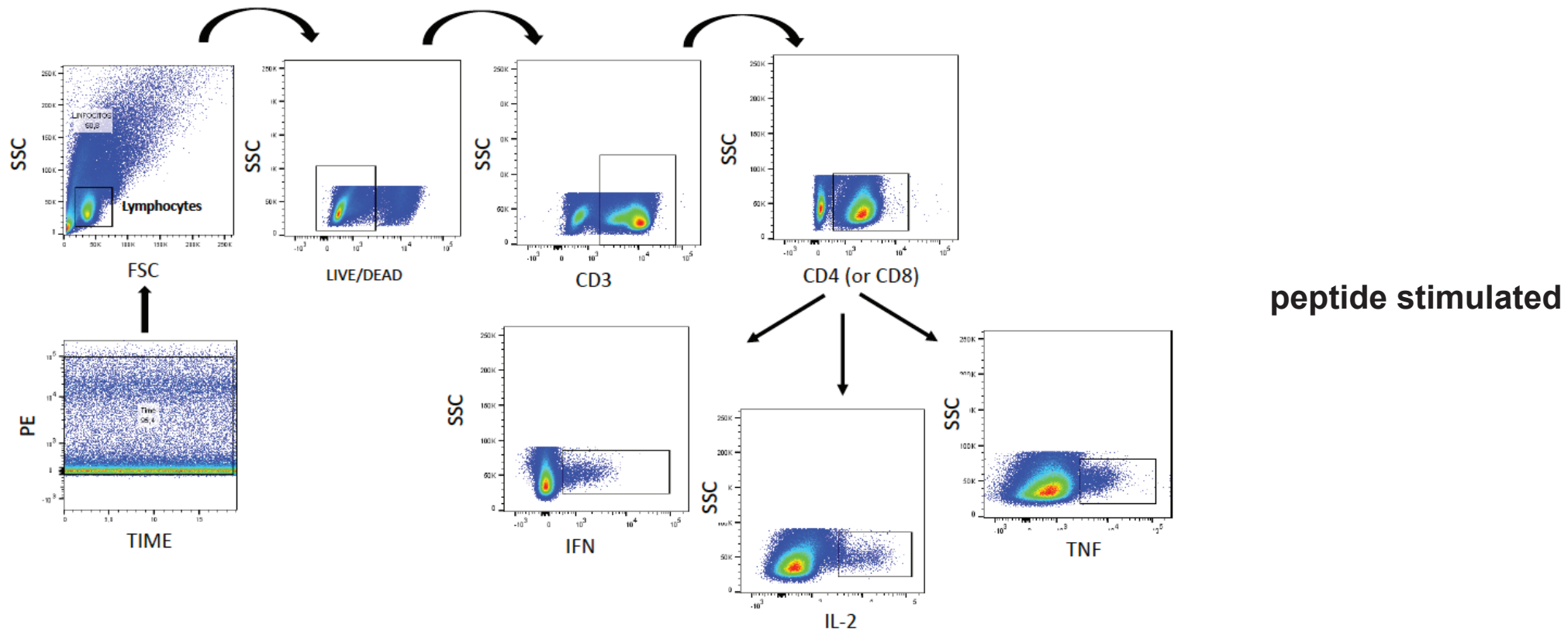

Supplement: Supplementary file 5 — Additional file 5: Fig. S5. Gating strategy employed to analyze cytokine production in T-lymphocytes. Panels A, B. Cytokine production was determined in cells left unstimulated (A) or stimulated ex-vivo with the cell therapy peptides (B). [file 12981_2021_426_MOESM5_ESM.pdf]

A

CD4+ T-cells

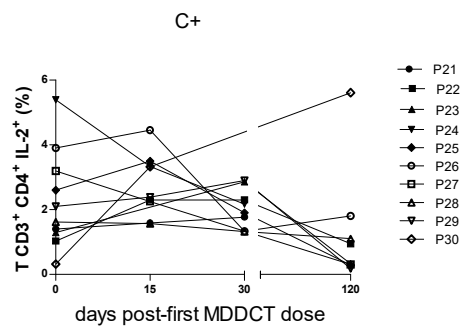

B

CD8+ T-cells

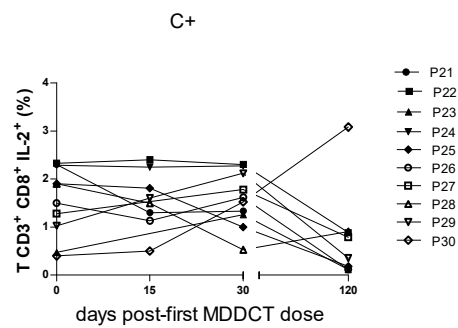

IL-2

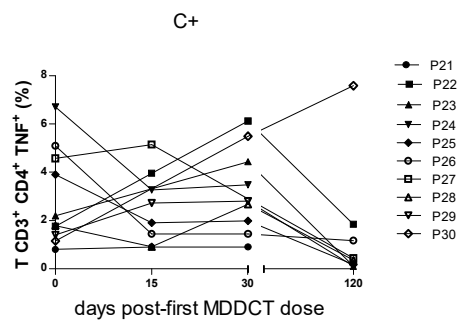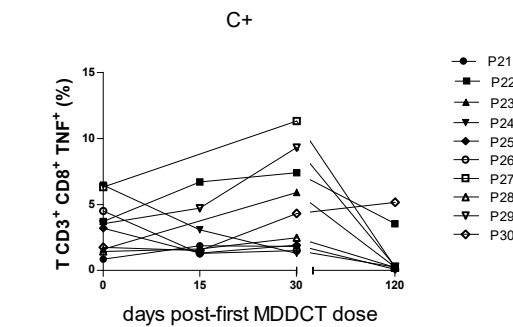

TNF

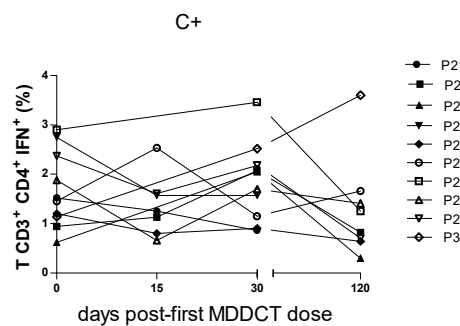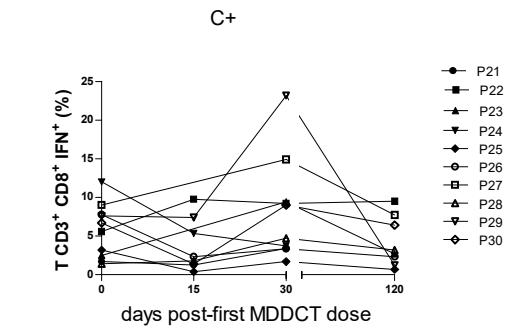

IFN

Supplement: Supplementary file 6 — Additional file 6: Fig. S6. Cytokine expression in SEB/brefeldin-stimulated CD4+ and CD8+ T-lymphocytes following MDDC MDDCT. Data were obtained as described in Fig. 3. [file 12981_2021_426_MOESM6_ESM.pdf]

A

CD4+ T-cells

(unstimulated)

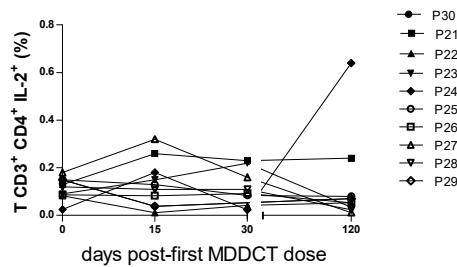

B

CD8+ T-cells

(unstimulated)

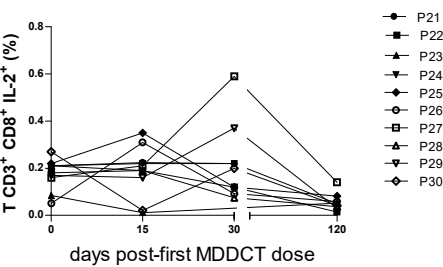

IL-2

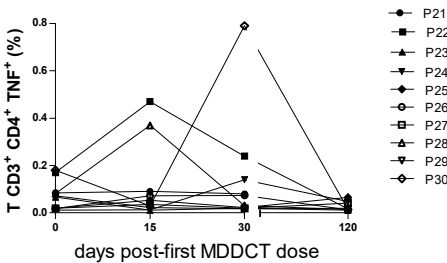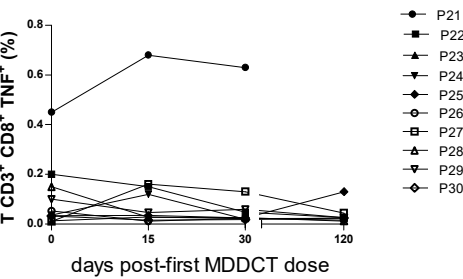

TNF

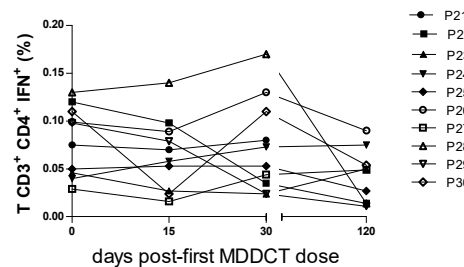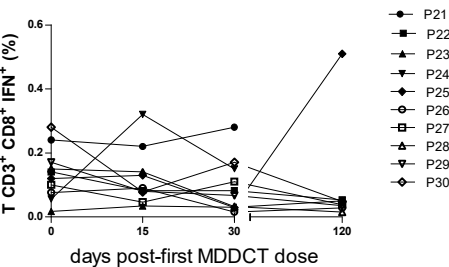

IFN

Supplement: Supplementary file 7 — Additional file 7: Fig. S7. Cytokine expression in unstimulated CD4+ and CD8+ T-lymphocytes following MDDC MDDCT. Data were obtained as described in Fig. 3. [file 12981_2021_426_MOESM7_ESM.pdf]

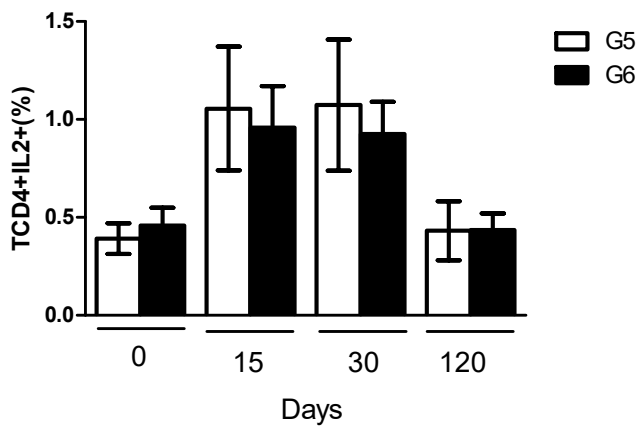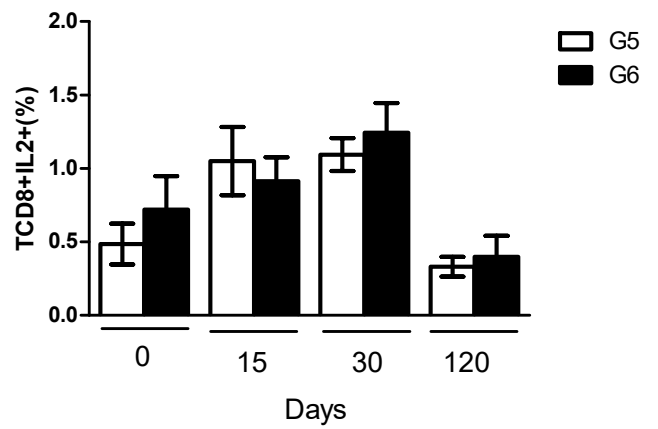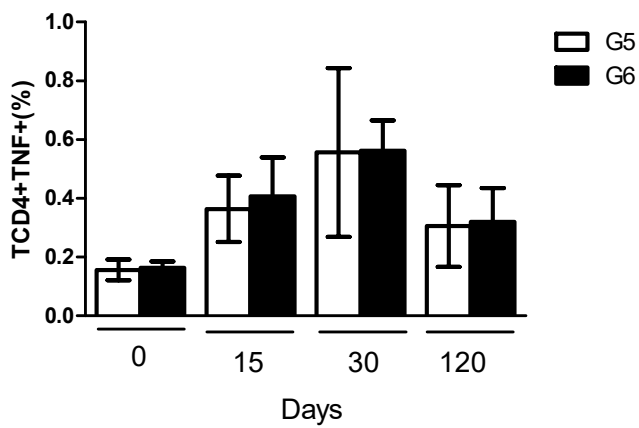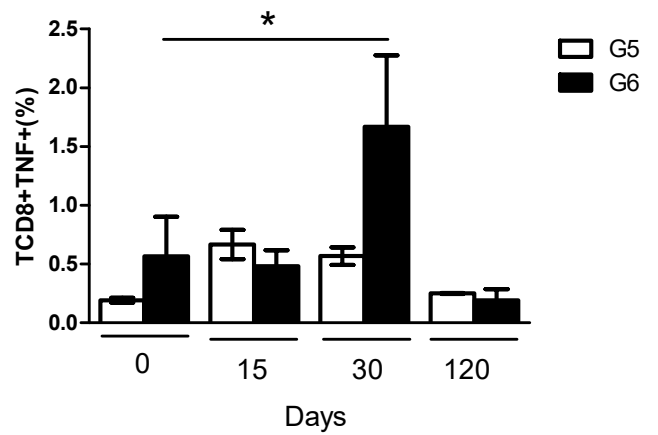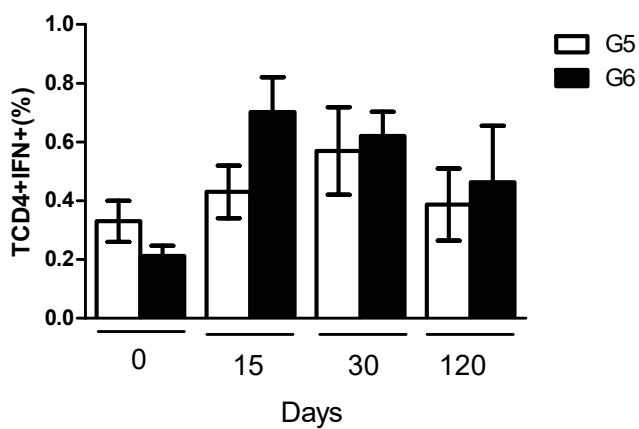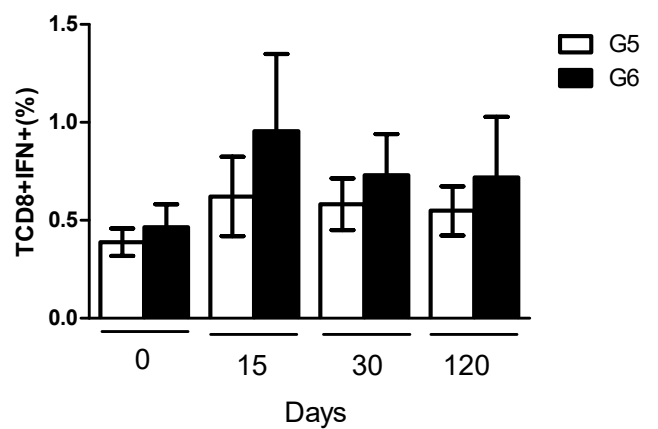

Supplement: Supplementary file 8 — Additional file 8: Fig. S8. Inter-group comparison of the immunogenicity of MDDCT. Comparison of the levels of IFN-γ, IL-2, and TNF production levels by CD4+ and CD8+ T-cells in individuals receiving MDDCT only (G5) and individuals receiving MDDCT with nicotinamide and auranofin (G6). Data were analyzed by one-way ANOVA followed by Dunn´s post-test. *p < 0.05. [file 12981_2021_426_MOESM8_ESM.pdf]

Time and type of treatment:  
Cumulative  $P = 0.07$

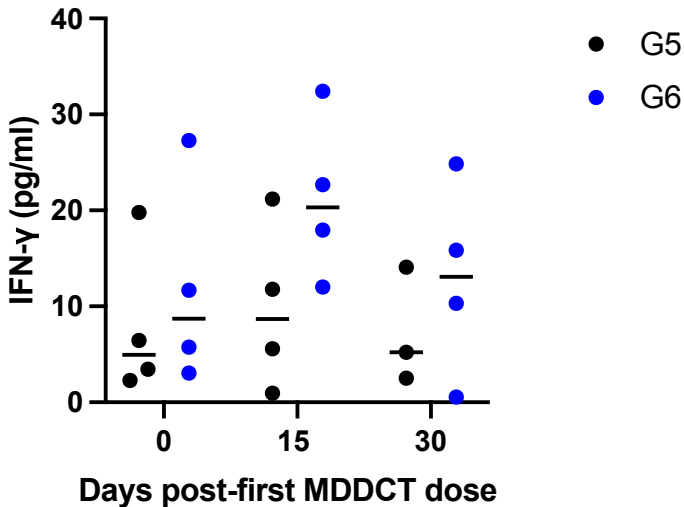

Supplement: Supplementary file 9 — Additional file 9: Fig. S9. Impact of MDDCT on the level of IFN- γ in plasma. The Fig. shows the level of IFN-γ in individuals receiving the personalized dendritic-cell therapy (DCT), alone (G5, in black) or following the conditioning regimen consisting of auranofin + nicotinamide (G6, in blue). IFN-gamma was measured by ELISA testing, and data were analyzed by two-way ANOVA. The P-value reported refers to the interaction between time and the previous conditioning regimen. [file 12981_2021_426_MOESM9_ESM.pdf]

**A**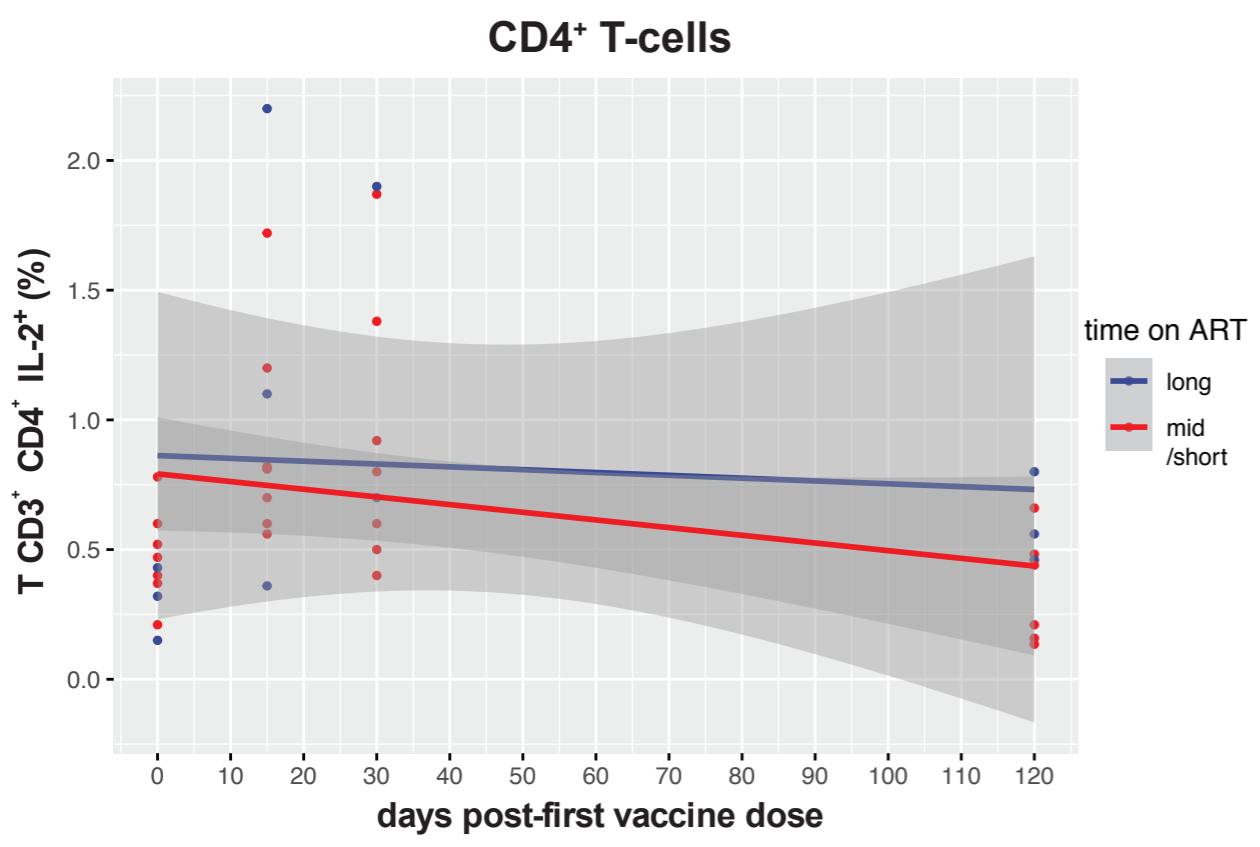**B**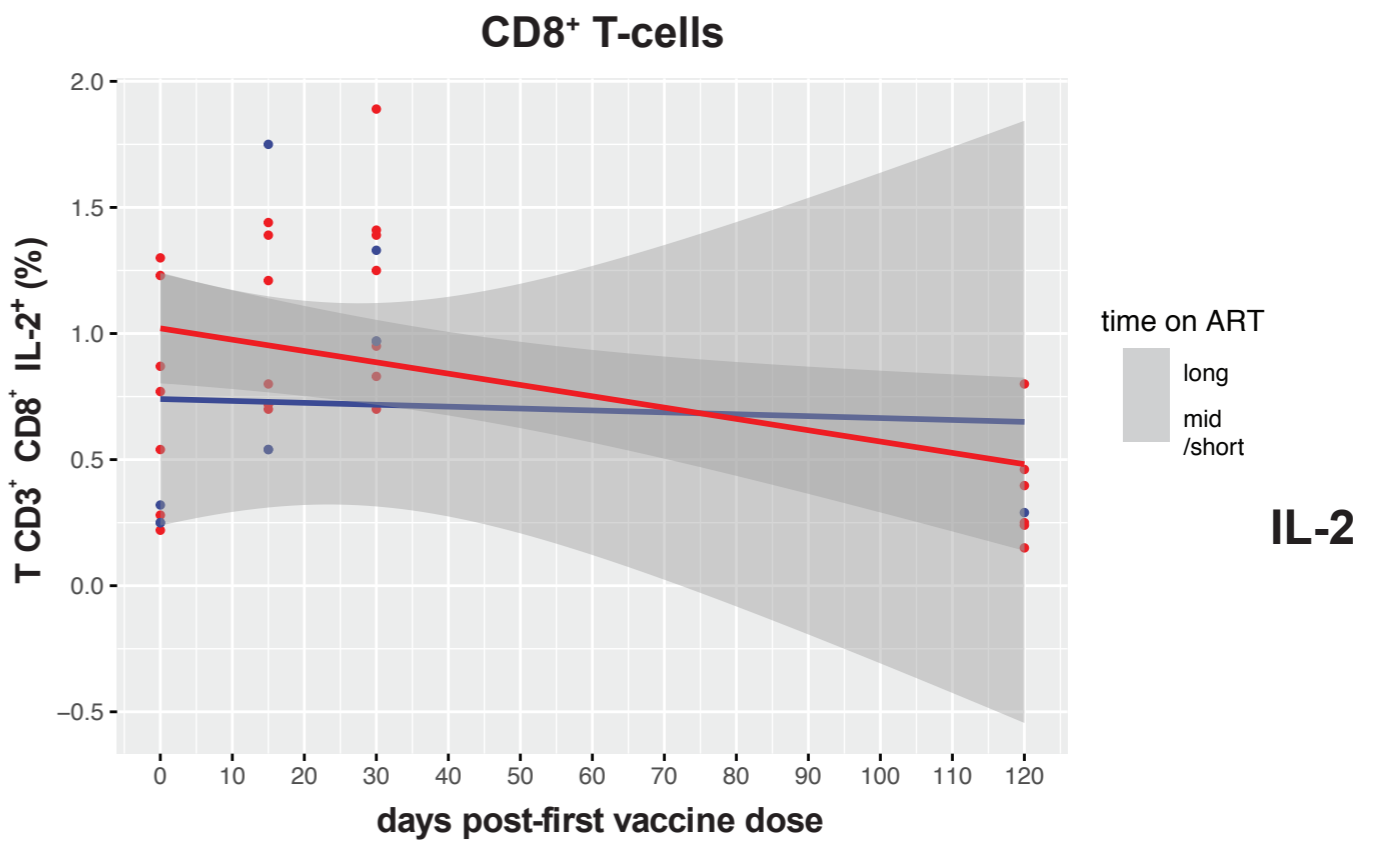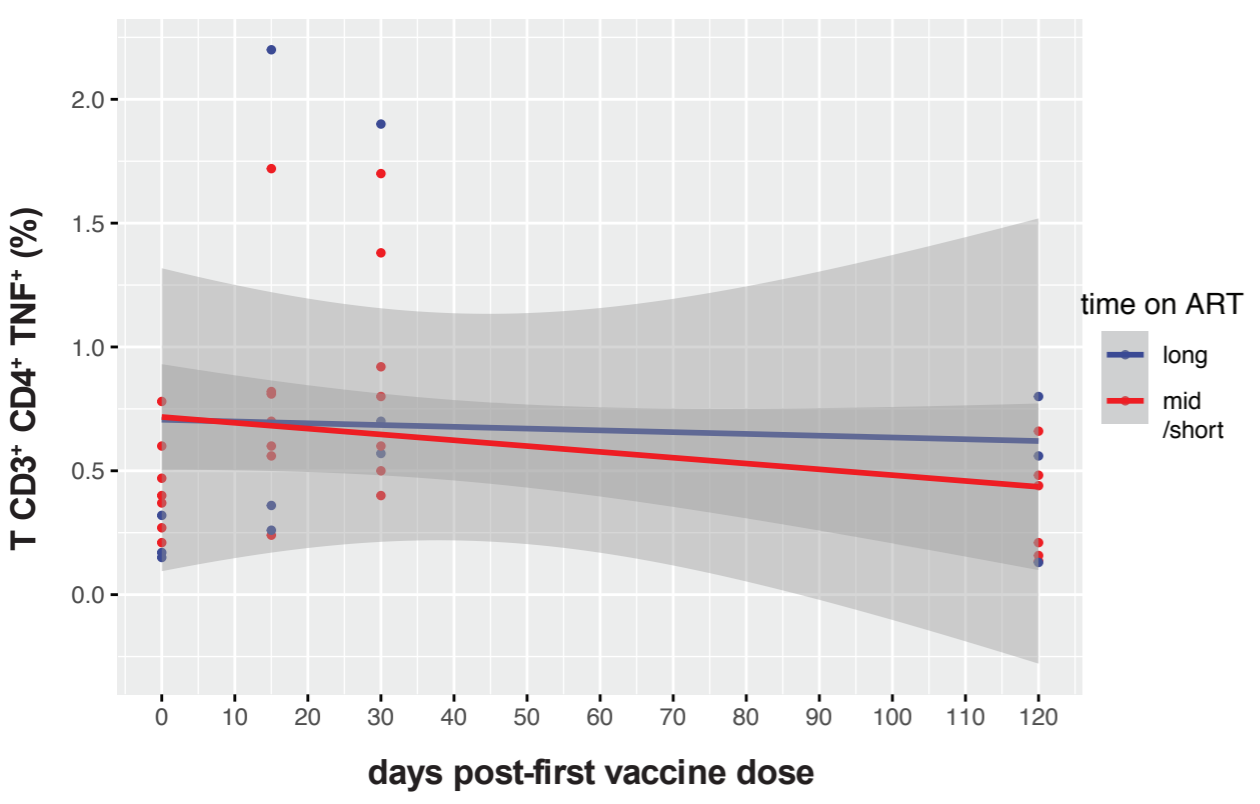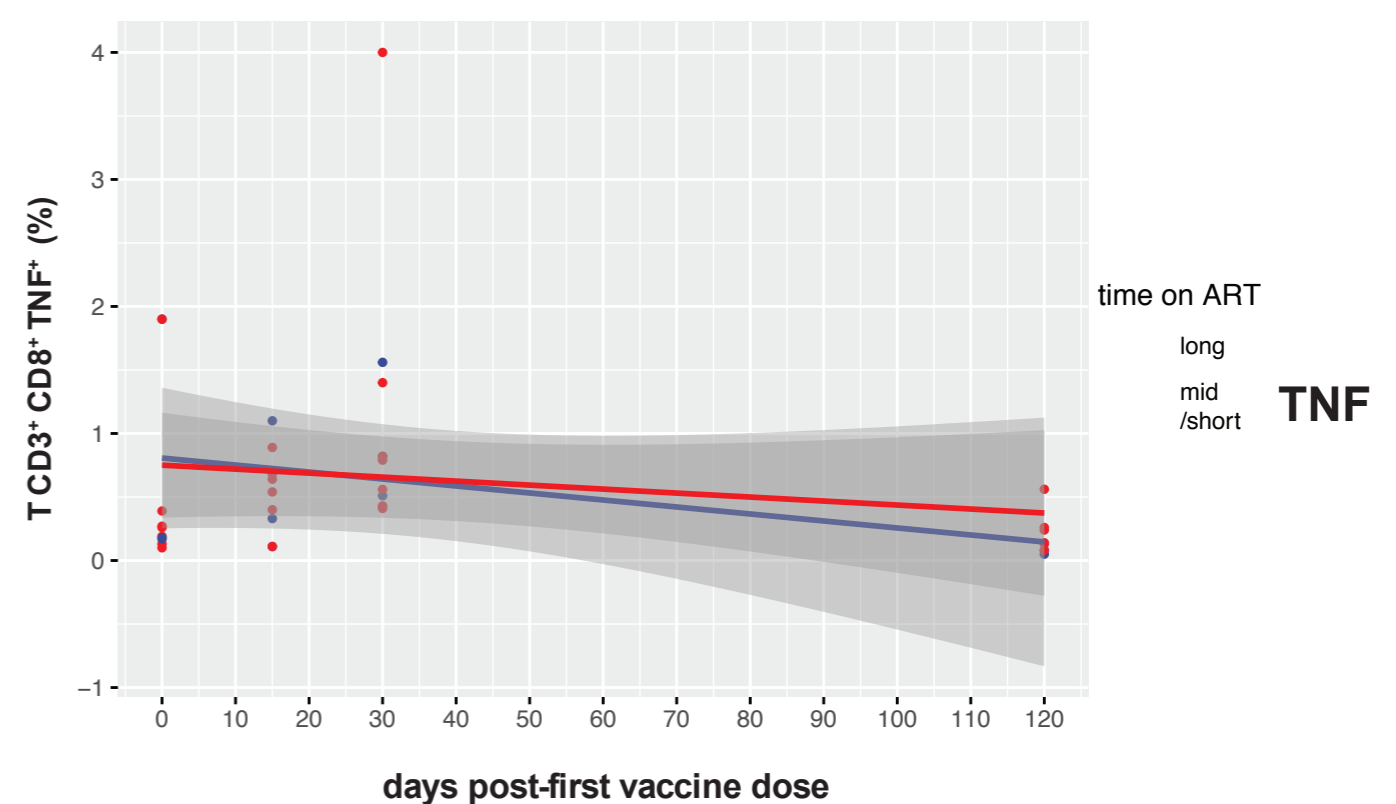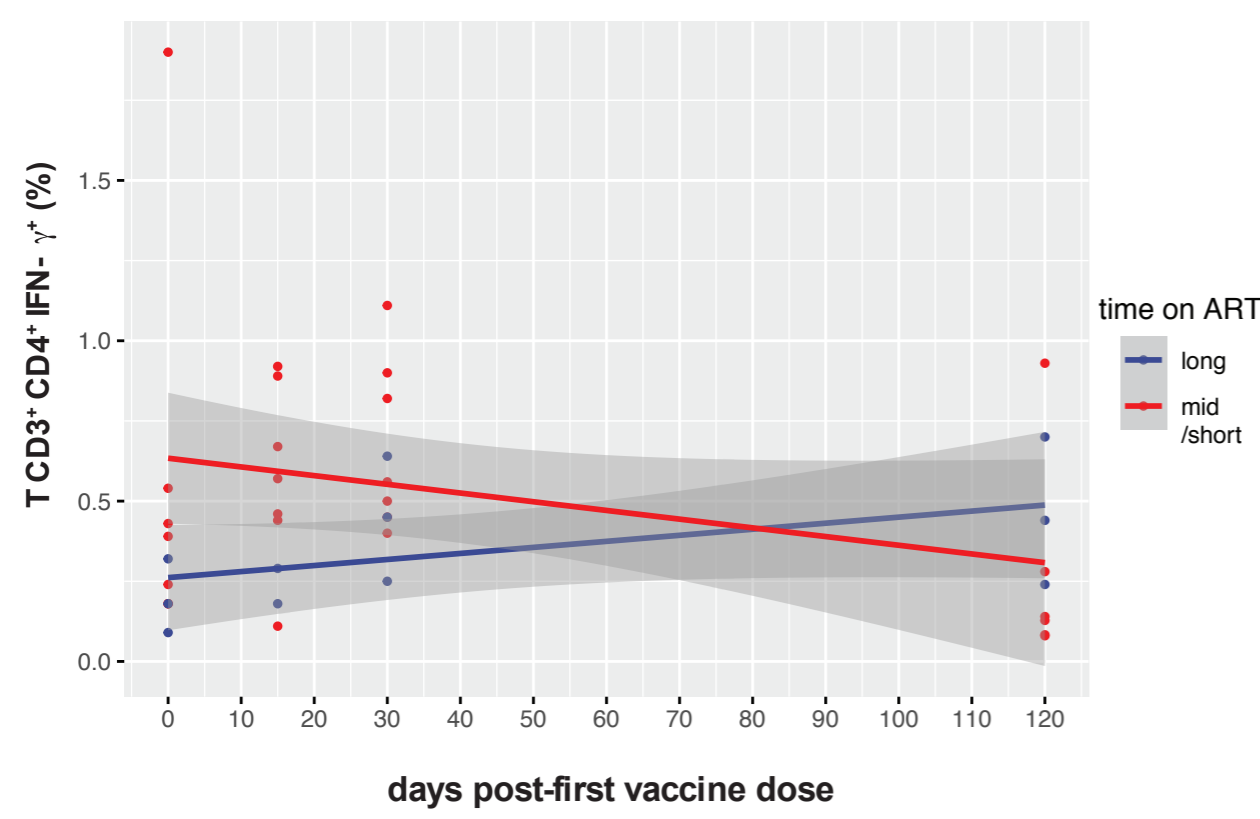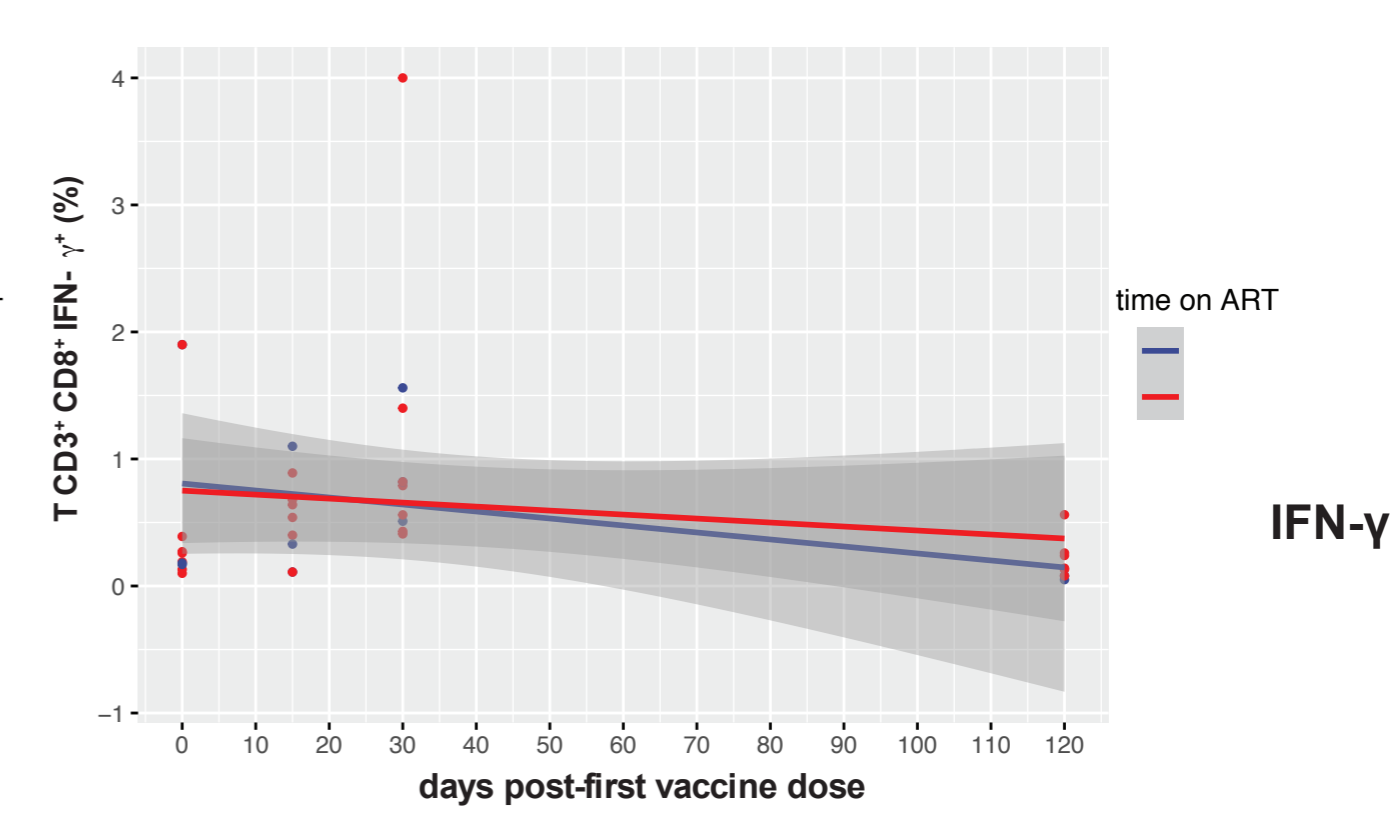

Supplement: Supplementary file 10 — Additional file 10: Fig. S10. Impact of the time on ART on cytokine production post-MDDCT. The graph visualizes the relation between the time in days since the first MDDCT dose and the expression of immune response mediating cytokines in CD4+ (Panel A) and CD8+ (Panel B) T-cells. Patients were stratified into two categories based on the overall time of ART administration since their diagnosis (i.e. > 10 years, “long” and < 10 years, “mid/short”). The lines on the graph represent linear regression slopes for each group, and the gray areas indicate 95% confidence intervals for each line. [file 12981_2021_426_MOESM10_ESM.pdf]
