# Supplementary material for: Immunogenicity of personalized dendritic-cell therapy in HIV-1 infected individuals under suppressive antiretroviral treatment: interim analysis from a phase II clinical trial
Source: AIDS Res Ther. 2022 Jan 12;19:2. doi: 10.1186/s12981-021-00426-z (PMC8753935; doi:10.1186/s12981-021-00426-z)
Supplement: Supplementary file 2 — Additional file 2: Fig. S2. Representative example of the viability of MDDCs under different maturation protocols and conditions. MDDCs were isolated from PBMCs and induced to maturation according to two protocols based on IL-4 and IFN-α as depicted in Additional file 1: Fig. S1. Maturation of MDDCs was performed starting from either freshly collected PBMCs (red bars) or PBMCs frozen in liquid nitrogen upon collection and thawed after 2 weeks. Viability was assessed by flow cytometry using a LIVE/DEAD stain. Data were normalized over the fresh-cell condition. [file 12981_2021_426_MOESM2_ESM.pdf]

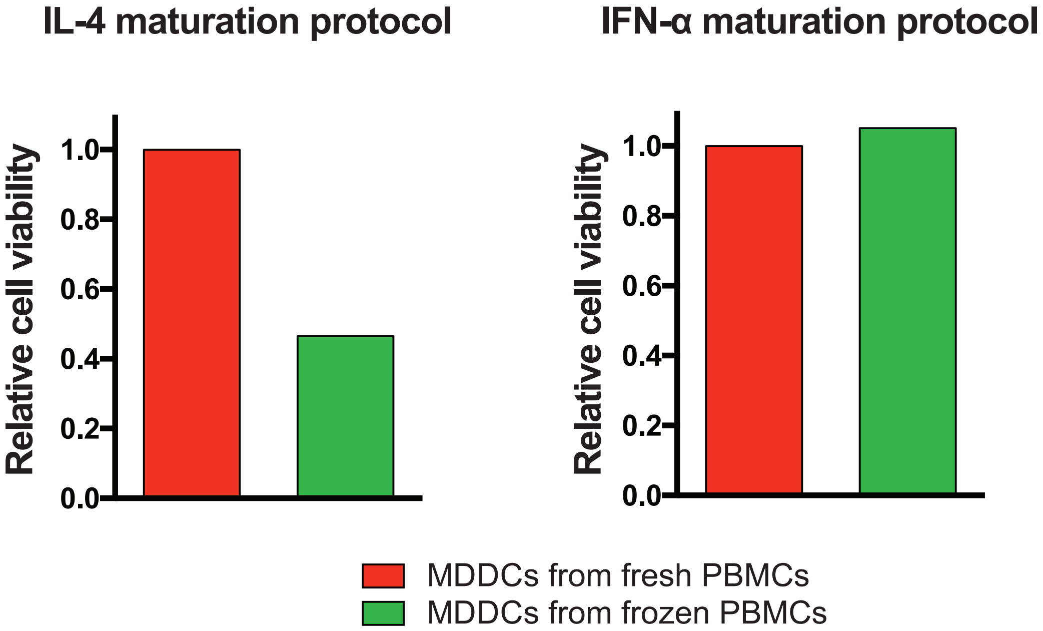
IL-4 maturation protocol
IFN-α maturation protocol
Relative cell viability
1.0
0.8
0.6
0.4
0.2
0.0
Relative cell viability
1.0
0.8
0.6
0.4
0.2
0.0
MDDCs from fresh PBMCs
MDDCs from frozen PBMCs
