# Supplementary material for: Immunogenicity of personalized dendritic-cell therapy in HIV-1 infected individuals under suppressive antiretroviral treatment: interim analysis from a phase II clinical trial
Source: AIDS Res Ther. 2022 Jan 12;19:2. doi: 10.1186/s12981-021-00426-z (PMC8753935; doi:10.1186/s12981-021-00426-z)
Supplement: Supplementary file 11 — Additional file 11: Table S1. List of peptides used for pulsing MDDCs of each individual. Enneamers (9 mers) were designed according to their predicted immunogenicity in a given individual, as described in the main text. Peptides were then used to pulse MDDCs before their reinfusion in MDDCT recipients according to the workflow depicted in Additional file 1: Fig. S1. Note that some autologous peptides are common to more than one patient. [file 12981_2021_426_MOESM11_ESM.docx]

| Patient ID | Gag vaccine peptides |
| --- | --- |
| P21 | **WIILGLNKI**; **YVDRFYKTL; KALGPAATL** |
| P22 | **VHEKKEVRD; KEVRDTKEA; TIKCFNCGK; GPKRTIKCF; TLYCVHEKK; CVHEKKEVR** |
| P23 | **PEVIPMFSA; FSPEVIPMF** |
| P24 | **WIILGLNKI; GLNKIVRMY; FRDYVDRFY; RAEQASQEV** |
| P25 | **VGEIYKRWI; WIILGLNKI; QNANPDCKTI; VKNWMTETL** |
| P26 | **WIILGLNKI**; **YVDRFYKTL; KALGPAATL** |
| P27 | **VLAEAMSKV; GPGHKARVL** |
| P28 | **KVKNMTESL; LRLNKIVRM; AEWDRLHPV** |
| P29 | **VLAEAMSQV; QVTNATTVM; KLNPYIIQY; YIIQYQPLY; IQYQPLYCV; QKIEVRDTK** |
| P30 | **WIILGLNKI**; **YVDRFYKTL; KALGPAATL** |
